# Supplementary material for: Co-designing an intervention to increase uptake of advance care planning in later life following emergency hospitalisation: a research protocol using accelerated experience-based co-design (AEBCD) and the behaviour change wheel (BCW)
Source: BMJ Open. 2022 May 18;12(5):e055347. doi: 10.1136/bmjopen-2021-055347 (PMC9121419; doi:10.1136/bmjopen-2021-055347)
Supplement: Supplementary data [file bmjopen-2021-055347supp001.pdf]

Supplementary appendix

Co-designing an intervention to increase uptake of Advance Care Planning (ACP) in later life: Accelerated experience-based co-design (AEBCD) online workshop outline

Prior to online workshop

- Participants register beforehand by accepting Microsoft Teams invites (via email) and returning consent form (via e-mail).

- Participants complete brief questionnaire about their background & readiness towards ACP via email.

- Participants informed via email that anyone can take a break whenever they need and if the workshop raises any emotional concerns in people, they are welcome to speak to a mental health first aider after the meeting

- Participants encouraged to log-in early to workshop if possible (up to 15 mins beforehand if desired to check for any technical issues e.g. WiFi connectivity)

- Recognition within the facilitator and co-design team that an element of flexibility in the schedule may be required on the day to respond to participants' needs and contributions during the live workshop.

Resources needed for workshop sessions

- Internet access
- Microsoft Teams software (including breakout rooms, electronic chat on Teams)
- PowerPoint (using with share screen function on Microsoft Teams)
- Software for interactive answers, prioritisation and voting e.g. Mentimeter or equivalent
- Notepad to take additional field notes or use of virtual white board e.g. Jamboard
- Feedback forms

Persons involved in both sessions

- Principal investigator (to act as main facilitator)
- Co-investigators (to act as assistant facilitators)
- Participants: 12 in total (consisting of 4 older persons, 4 carers and 4 healthcare professionals). An extra healthcare professional may need be invited in case of dropouts on the day of the workshop to balance the number of participants.

Role designation of workshop team to facilitate discussion

Role of the main facilitator

The discussion will be facilitated by a main facilitator presenting the content of previous research and asking participants questions about their experiences and views, with an emphasis on open questioning, asking probing questions where appropriate and clarifying participants' comments.
The main facilitator will also answer any other verbal questions that participants may have during the workshop, particularly related to ACP and behaviour change.

Role of the assistant facilitators

In order to focus on presentation of content for the discovery phase and driving the verbal discussion during the workshop, the main facilitator will be assisted by two assistant facilitators for technical support, note-taking and timekeeping.

The first assistant facilitator will take notes, attend to any technical software questions, and field text group chat questions in the Microsoft Teams interface, with the support of the second facilitator on this latter task if necessary.

The second assistant facilitator will focus on time-keeping of the workshop, and ensuring all voices are heard and considered, including keeping track of which participants have spoken, which participants wish to further contribute, including noting the order of participants "raising hands" on Microsoft Teams.

Timetable A: Half-day session 1 of 2 -DISCOVERY PHASE (Duration ~ 2 hours, 25 mins)

Activity	Description	Format	Deliverables	Time
Welcome & introduction	• Aims of workshop & brief agenda *(10mins)*<br><br>• Recap that both experts in experience and clinicians are participating in the workshop<br><br>• Information given to participants that anyone can take a break whenever needed and if the workshop raises any emotional issues, they are welcome to speak to a mental health first aider<br><br>• Icebreaker e.g., names, book/TV show/hobby enjoyed in lockdown and brief explanation of motivation to take part in the workshop *(5mins)*	Microsoft Teams	Responses on motivations to take part	~15mins
Discovery phase	• Overview of what Advance Care Planning (ACP) is *(5mins)*<br><br>• Investigator(s) sharing results of previous projects exploring views of ACP in older adults of our different stakeholder groups (patients, carers, general public and healthcare professionals) *(20mins)*<br><br>• Reflection and discussion of different experiences, views and what informs ACP in later life, including amongst different stakeholder groups *(conducted in 2 smaller groups consisting of 2 older persons, 2 carers and 2 healthcare professionals, who can verbalise and use chat function of Teams, followed by a discussion in the main room with a 2-minute summary by each facilitator of the discussion) (25mins)*<br><br>*Comfort break (10 mins)*<br><br>• Discussion (*in breakout rooms with feedback to full group)*: Quick recap on ACP beforehand. Warm up question: Have you had experience in ACP? Why is ACP not more widely taken up? Discussion of ACP as a behaviour & barriers & facilitators *(20 mins)*<br><br>• Introduction to Behaviour Change Wheel (behaviour theory) *(5mins)*	PowerPoint<br><br>Guided Q & A<br><br>Open discussion<br><br>Nominal group technique<br><br>Small group & large group idea generation<br><br>Mentimeter poll	Responses from participants for<br><br>1) experiences & views on ACP<br><br>2) barriers & facilitators of ACP	~1 hour 30 mins

Preparation for co-design phase	• What would be useful to end-users to co-design? – reflection of useful initiatives and discussion/voting/decision. Use assessment to the process of developing, selecting and implementing behavioural interventions using the acronym APEASE **A**cceptability, **P**racticability, **E**ffectiveness, **A**ffordability, **S**ide-effects and **E**quity - (Public Health England, 2020) *(25mins)*<br><br>• Summarise concept for co-design ahead of session 2 *(5mins)*	PowerPoint<br><br>Q & A<br><br>Open discussion<br><br>Nominal Group Technique<br><br>Mentimeter poll	Responses and decision from participants for what to co-design	~30 mins
Conclusion & summary	• Conclusion/summary for first session<br><br>Next steps:<br>• Opportunity for feedback -contact details given for e-mail feedback if wished and mental health first aider details if the workshop has raised any difficult emotions for any participant.<br><br>• Encouragement to informally reflect on design between sessions (optional)<br><br>• Payment for participants (done via e-mail if wished)	PowerPoint	Feedback comments	~10 mins

Timetable B Half-day session 2 of 2 -CO-DESIGN PHASE USING THE BEHAVIOUR CHANGE WHEEL (Duration ~ 2 hours, 30 mins)

Activity	Description	Format	Deliverables	Time
Welcome back & recap	• Welcome back & recap to the workshop *(5mins)*<br>• Inform participants that anyone can take a break whenever they need and if the workshop raises any emotions, they are welcome to speak to a mental health first aider after the meeting<br>• Ice-breaker -re-introduce names *(5 mins)*<br>• Any comments/questions *(5mins)*	Microsoft Teams<br><br>PowerPoint		~15 mins
Co-design phase	• Recap on Behaviour Change Wheel and the process of developing, selecting, and implementing behavioural interventions using the acronym APEASE to drive the discussion - **A**cceptability, **P**racticability, **E**ffectiveness, **A**ffordability, **S**ide-effects and **E**quity -(Public Health England, 2020). *(10 mins)*<br>• Questions regarding the APEASE acronym (5 mins during/after discussing the APEASE criteria)<br>• Prioritising important values and behaviours for ACP in later life to different stakeholder groups in relation to Behaviour Change Wheel (patients, carers, clinicians) *(20 mins)*<br>• Co-design activity for an intervention to increase uptake ACP in older adults to guide social contacts and professionals using the Behavioural Change Wheel Method -consider the context/setting, format *(25 mins)*<br>*Comfort break 10 mins*<br>• Co-design activity continued, focusing on content (including detail) and linguistic approach of the intervention. *(25 mins)*<br>• Summary of intervention -overview, review using APEASE criteria, refinement and reflection on co-design activity *(20mins)*<br>• Discuss how this might be applied to policy *(10 mins)*	Guided Q & A<br><br>Open discussion<br><br>Nominal group technique<br><br>Ideas discussed in small groups with later feedback to larger groups<br><br>Selected Mentimeter poll	Responses on values & behaviours<br><br>Co-designed product - information on context, format, content and linguistic approach<br><br>APEASE criteria feedback<br><br>Ideas on policy applications	~2 hours

Conclusion	• Summary and thanks<br>• Opportunity for feedback, including details for mental health first aider in case the workshop has raised any difficult emotions<br>• Participants invited for voluntary follow-up call or email 4 weeks after the study to check readiness towards ACP<br>• Inform participants regarding forthcoming optional update about results/output of the study if participants agree			~15 mins

Co-designing an intervention to facilitate future care planning behaviours in later life

Participant questionnaire

What is your age? _________

1) **What is your gender?** *(Please tick)*
   - *Male*
   - *Female*
   - *Other (please specify)* ___________________________
   - *Prefer not to say*
2) **Please let us know about your background** *(please tick all that apply)*
   - I have experience of being a patient
   - I have experience of being a carer for an older person (aged 50+)
   - I am a healthcare professional

If you are a healthcare professional, please tell us about your role

Type of healthcare professional (please specify e.g. nurse)

____

Specialty (please specify) _________________________

Seniority (please specify) _________________________

Years working in healthcare (please specify) __________

Please feel free to tell us more about yourself in the space below
(e.g. your own experience of being a patient, caring or healthcare work, including any experience with emergency admissions to hospital).

3) ***How do you feel about future care planning for yourself?*** *(Please tick which statement best describes you)*

- *I have no need to plan my future care and I am not interested in this for myself whatsoever.*

- *I am aware future care planning might be relevant, but I am not planning to make a future care plan at this stage.*

- *I am planning on finding out more about future care planning and preparing to take the first steps to make a future care plan.*

- *I am in the process of making a future care plan for myself.*

- *I have made a future care plan for myself.*

- *I was interested in future care planning and/or had a future care plan but since then have changed my mind.*

If you are happy to be contacted 1 month after the study to be asked if you feel the same way about future care planning, please write your preferred contact details *(e.g. email/phone number).*
