# Supplementary material for: Co-designing an intervention to increase uptake of advance care planning in later life following emergency hospitalisation: a research protocol using accelerated experience-based co-design (AEBCD) and the behaviour change wheel (BCW)
Source: BMJ Open. 2022 May 18;12(5):e055347. doi: 10.1136/bmjopen-2021-055347 (PMC9121419; doi:10.1136/bmjopen-2021-055347)
Supplement: Author's manuscript [file bmjopen-2021-055347.draft_revisions.pdf]

BMJ Open is committed to open peer review. As part of this commitment we make the peer review history of every article we publish publicly available.

When an article is published we post the peer reviewers' comments and the authors' responses online. We also post the versions of the paper that were used during peer review. These are the versions that the peer review comments apply to.

The versions of the paper that follow are the versions that were submitted during the peer review process. They are not the versions of record or the final published versions. They should not be cited or distributed as the published version of this manuscript.

BMJ Open is an open access journal and the full, final, typeset and author-corrected version of record of the manuscript is available on our site with no access controls, subscription charges or pay-per-view fees (http://bmjopen.bmj.com).

If you have any questions on BMJ Open's open peer review process please email info.bmjopen@bmj.com

BMJ Open

Co-designing an intervention to increase uptake of Advance Care Planning in later life following emergency hospitalisation: a research protocol using Accelerated Experience-Based Co-design (AEBCD) and The Behaviour Change Wheel (BCW).

Journal:	*BMJ Open*
Manuscript ID	bmjopen-2021-055347
Article Type:	Protocol
Date Submitted by the Author:	14-Jul-2021
Complete List of Authors:	Bielinska, Anna-Maria; Imperial College London, Department of Surgery and Cancer; Imperial College Healthcare NHS Trust<br>Archer, Stephanie; Imperial College London, Department of Surgery and Cancer; University of Cambridge, Department of Public Health and Primary Care<br>Darzi, Ara; Imperial College London, Department of Surgery and Cancer; Imperial College Healthcare NHS Trust<br>Urch, Catherine; Imperial College London Department of Surgery and Cancer; Imperial College Healthcare NHS Trust
Keywords:	GERIATRIC MEDICINE, PALLIATIVE CARE, ACCIDENT & EMERGENCY MEDICINE, Health policy < HEALTH SERVICES ADMINISTRATION & MANAGEMENT, QUALITATIVE RESEARCH, EDUCATION & TRAINING (see Medical Education & Training)

SCHOLARONE™
Manuscripts

TITLE PAGE

TITLE

Co-designing an intervention to increase uptake of Advance Care Planning in later life following emergency hospitalisation: a research protocol using Accelerated Experience-Based Co-design (AEBCD) and The Behaviour Change Wheel (BCW).

Lead & corresponding author

Dr Anna-Maria Bielinska[1,2], Clinical Research Fellow, Imperial College
Email: anna-maria.bielinska@doctors.org.uk
Postal address: Academic Surgical Unit, Department of Surgery and Cancer, 10th Floor QEQM Building, St Mary's Hospital, Praed Street, Paddington, London, W2 1NY

Co-authors:

Dr Stephanie Archer[1,3]
Professor Lord Ara Darzi[1,2]
Professor Catherine Urch[1,2]

Affiliations:

1) Department of Surgery and Cancer, Imperial College, London
2) Imperial College Healthcare NHS Trust, London
3) The University of Cambridge, UK

Word count 3294 words, excluding appendix (limit 4000 words, including abstract and strengths/limitations)

Planned study
Dates of study included in manuscript and cover letter

Ethical approval
obtained

For peer review only

Title:

Co-designing an intervention to increase uptake of Advance Care Planning in later life following emergency hospitalisation: a research protocol using Accelerated Experience-Based Co-design (AEBCD) and The Behaviour Change Wheel (BCW).

Abstract:

Introduction: Despite the potential benefits of advance care planning, uptake in older adults is low. In general, there is a lack of guidance as to how to initiate advance care planning conversations and encourage individuals to take action in planning their future care, including after emergency hospitalisation. Participatory action research methods are harnessed in health services research to design interventions that are relevant to end-users and stakeholders. This study aims to involve older persons, carers and healthcare professionals in co-designing an intervention to increase uptake of advance care planning in later life, which can be used by social contacts and healthcare professionals, particularly in the context of a recent emergency hospitalisation.

Methods and analysis: The theory-driven participatory design research method integrates and adapts Accelerated Experience-Based Co-Design with the Behaviour Change Wheel, in the form of a collaborative multi-stakeholder co-design workshop. In total, 12 participants, comprising 4 lay persons aged 70+, 4 carers and 4 healthcare professionals with experience in elder care, will be recruited to participate in two online half-day sessions, together comprising one online workshop. There will be a maximum of 2 workshops. Firstly, in the discovery phase, participants will reflect on findings from earlier qualitative research on views and experiences of advance care planning from three workstreams: patients, carers, and healthcare professionals. Secondly, in the co-design phase, participants will explore practical mechanisms in which older persons aged 70+ can be encouraged to adopt advance care planning behaviours based on the Behaviour Change Wheel, in order to co-design a behavioural intervention to increase uptake of advance care planning in older adults after an emergency hospitalisation.

Ethics and dissemination: Ethical approval has been obtained from the Science Engineering Technology Research Ethics Committee at Imperial College London (Reference:19IC5538). The findings from this study will be disseminated through publications, conferences and meetings.

For peer review only

Strengths and limitations of this study

1) The study draws upon previous research exploring the views of patients, carers and healthcare professionals in order to design a behavioural intervention.

2) Participants can reflect their own views and experiences during a highly collaborative co-design process, which recruits a diverse group of participants, including lay persons aged 70+, carers and healthcare professionals with experience in elder care.

3) To our knowledge this the first study to use the Behaviour Change Wheel to design a behavioural intervention to increase uptake of advance care planning in later life following an emergency hospitalisation.

4) To our knowledge this is also the first study combining Accelerated Experience Based Co-design with the BCW, other studies having used Experience Based Co-design over longer time frames.

5) The online workshop might digitally exclude older adults with limited access to the internet.

For peer review only

Introduction

Advance care planning (ACP) is a spectrum of approaches to planning future care (1) which "supports adults at any age or stage of health in understanding and sharing their personal values, life goals, and preferences" (2). ACP can be started at any point in an individual's life course, including during chronic and serious illness (3). It is an iterative process involving discussions between an individual and their care provider(s) and may cover a range of domains, from advance statements regarding care preferences, to advance decisions to refuse treatment (ADRT). Individuals may also decide to nominate a surrogate decision-maker in case of future loss of capacity (4). ACP has been highlighted as critical to supporting shared decision making between patients and healthcare professionals (1) through maintaining the autonomy and self-determination of patients. Research in older adults supports early engagement in ACP and highlights its importance in current and future care as this group may have multi-morbidities, complex psychosocial needs and face social isolation (5-7).

ACP occurs in a range of settings including the home, general practice, nursing homes, specialist hospital outpatient services and hospital wards (4). Traditionally, ACP was mainly pursued in palliative care (8), however there is increased recognition that older hospitalised adults, particularly those who have had an emergency hospitalisation, are an important cohort for ACP (1, 9, 10). As around one in five patients aged 70+ admitted to hospital as an emergency are in the last year of life (1), acute hospitalisation can act as a trigger for tailored ACP (1, 10). Qualitative research has shown that hospitalised older adults believe that ACP has a beneficial role in planning for their physical and psychosocial aspects of health, and patients look towards the collaborative expertise of clinicians to discuss and enact ACP (1). Furthermore, older inpatients also regard ACP as having a role in contemplating the possibility of physical deterioration, death and dying when considering healthcare choices in later life (1). Carers of older hospitalised adults regard ACP as process which helps to support older individuals, particularly in terms of the well-being, quality of life and independence (11).

Despite the potential benefits of ACP in older adults receiving emergency hospital care, such as improved concordance of care with patient's wishes, improved end-of-life care and better emotional well-being in relatives (12), and notwithstanding the numerous care planning initiatives (13) (14), the uptake of ACP is low (15, 16). This is even amongst cohorts of patients who are facing life-changing illness, such as older and seriously unwell medial inpatients (15) and those attending cancer clinics (16). As such, it is essential that the uptake of ACP is increased in all patient groups (17), particularly older adults (18).

Rationale for the current study

Since ACP can be defined as a health behaviour, behaviour change models can help guide the development of interventions to increase the uptake of ACP (18). The Medical Research Council recommends that identifying and developing a theoretical understanding is integral to developing complex interventions, including understanding the process of change, rationale, expected impact and the mechanisms by which change is to be achieved (19). Whilst a wide range of behaviour change frameworks exist (20), the Behaviour Change Wheel (BCW) has been cited as a recommended resource when designing interventions (21). The BCW is an evidence-based framework, which consists of a "behavioural COM-B system" at the hub, encircled by "intervention functions" and thereafter surrounded by "policy categories" (20) -see figure 1.

For peer review only

The COM-B system is a framework that can be used to understand a behaviour and focuses on capability, opportunity and motivation (20, 21). There are nine intervention functions: education, persuasion, incentivisation, coercion, training, restriction, environmental restructuring, modelling and enablement (20). The policy categories at the rim of the wheel support the delivery of the intervention (20). The BCW has been used to guide the development of a range of interventions in both physical and mental health (22, 23). Specifically in older adults' healthcare, the BCW has been used in a variety of behavioural interventions, from improving exercise participation for older adults in the community (24), to strengthening the role of nurses in stroke rehabilitation services(25), altering medication prescribing practices for older adults (26) and preventing pressure ulcers in nursing homes (27). However, to date there are no published studies using the BCW to increase uptake of ACP in later life in any setting.

Whilst the BCW may be an appropriate and effective theoretical model on which to base the 'active ingredients' of an intervention, other approaches for their design and delivery must be considered, in order to deliver an intervention that is acceptable to those who will use it and/or implement it in their services. One such method is Experience Based Co-design (EBCD), which has been evaluated as an effective strategy for quality improvement in healthcare services through "narrative-based, participatory action research" (PAR) with patients, families and staff (28). Recent robust theory driven approaches have combined EBCD and the BCW to guide complex intervention development co-design, involving service users and other stakeholders (23). Both EBCD and the BCW are complementary and have been integrated in qualitative research (23). The stepwise, reflective and pragmatic approaches of both EBCD and BCW approaches help to provide solutions to benefit end-users in quality improvement initiatives (20, 28). EBCD involves six sequential steps (see figure 2).

Whilst the original ECBD format can vary in the length of time required to complete the process, typically lasting 6-12 months and occasionally exceeding this (29), a more expedited version is available (28). In *accelerated* experience-based co-design (AEBCD), pre-existing collected narratives of experience from relevant stakeholders such as patients and staff form the initial data to start co-design whilst maintaining the "rigorous and effective patient-centred quality improvement approach" (28).

Summary and aims

There is increasing evidence that an emergency admission may herald the last year of life in a subset of older adults (1, 10); increased uptake of ACP may be beneficial in this group. In order to address this need, the aim of this study is to use the BCW to co-design a behavioural intervention to increase uptake of ACP in later life after an emergency hospitalisation, through AEBCD with older persons, carers and healthcare professionals.

For peer review only

Methods and analysis:
Study design

The research study will use Accelerated Experience-Based Co-Design (AEBCD) within an online workshop (split into two sessions) to co-design of an intervention to increase the uptake of ACP in the over 70s. In line with AEBCD methodology (28), the qualitative data collected from earlier studies (see box 1) will be used as a starting point.

Box 1: Prior work informing the design of this study

In preparation for this study, the experiences and views of older patients, carers and healthcare professionals of ACP in later life had been collected via qualitative interviews (1, 11, 30). Across three qualitative interview studies, a total of 48 participants took part - 20 hospitalised older adults, 8 carers and 20 frontline healthcare professionals. Interviews with older adults aged 70+ admitted to hospital have shown that they believe that ACP is relevant to their care following an emergency hospitalisation across a spectrum of prognoses and that they look towards the expertise of healthcare professionals after a medical emergency to plan their future care collaboratively (1). Carers of older adults who have been hospitalised believe that ACP has a role in supporting individuals in later life and that enabling the ACP process is crucial through practical support mechanisms (11). Interviews with frontline healthcare professionals have shown that open conversations with patients regarding ACP are valued in guiding clinicians' decisions and that there is a role in educating the clinical workforce to better understand how to approach ACP (unpublished data).

The AEBCD workshop will be divided into two phases: an initial discovery phase and a second co-design phase. The discovery phase will present the previously collected data on ACP. Workshop participants will be asked to reflect and discuss any experiences of and views on ACP. This phase of the workshop will allow the group to identify and develop potential improvements to increase uptake of ACP (see session 1, timetable A).

The co-design phase will draw on the steps for intervention design using the BCW ((22); See Figure 3).

For peer review only

Whilst the BCW highlights the range of potential mechanisms which could be form the theoretical basis of our intervention, data from the prior qualitative research studies and wider literature has highlighted that increasing motivation via education may be efficacious in increasing uptake of ACP (1, 11, 31). As such, this stage of the codesign workshop will focus on developing the context/setting, format and content of an educational resource. This will be achieved through prioritising important values and behaviours for ACP in later life, and will consider the **a**cceptability, **p**racticability, **e**ffectiveness, **a**ffordability, **s**ide-effects and **e**quity (APEASE; PHE 2020) of any intervention (see timetable B).

Ethics:

Ethical approval has been obtained from the Science Engineering Technology Research Ethics Committee (SETREC) at Imperial College London (SETREC Reference: 19IC5538).

Study setting

The study will be conducted online on Microsoft Teams hosted by Imperial College.

Study participants

The AEBCD workshop will comprise of approximately 12 participants as follows:

- Four lay persons with experience of being a patient aged 70+ (ideally who have experienced an emergency admission)
- Four lay persons with experience of being a carer of an older person (particularly for those aged 70+)
- Four health and social care professionals with experience of caring for older patients aged 70+

Sampling method

The study will be advertised to lay persons via the Imperial Research Partners Group, Patient and Public Involvement initiatives and third sector voluntary. Lay persons interested in participating can contact the study team for more information. Healthcare professionals will be invited from Imperial College and other academic institutions through emails to existing contacts who have experience of caring for older persons. Those who wish to participate will be able to respond by email to the study team.

Where possible, purposive sampling will be used to achieve a diversity of lay people in terms of conditions and different experiences of ACP. Similarly, a purposive sample of healthcare professionals will be composed from different work backgrounds to enrich the study.

Participant inclusion criteria

The participant inclusion criteria are as follows:

- Participants must be able and willing to participate in a group discussion.
- Participants must have experience of being a patient, caring or are a healthcare professional with experience of older person's healthcare.
- Lay persons with experience of being a patient must be aged 70yrs+
- Carers and healthcare professionals must be aged any age 18yrs+

For peer review only

- Participants must have capacity to give informed consent
- Participants must be able to speak, read and write in English

Participant consent

Consent to enter the study will be sought from each participant after a full explanation has been given, a participant information sheet offered and time allowed for consideration (at least 24 hrs). Signed participant consent will be obtained. The right of the participant to refuse to participate without giving reasons will be respected and all participants will be free to withdraw at any time. If participants have already taken part in the workshop, their contribution will not be withdrawn as it contributes to a wider discussion, but no extracts from their contributions within the discussions will be used any write up or presentation.

Study procedures

Terminology used
Some researchers use the term "future care planning" instead of "advance care planning" as this is more intuitively understood (32). Future care planning will be used in this study with participants to aid the flow of the discussion.

Baseline questionnaire
Data will be individually gathered on basic demographic information such as age, sex and background (e.g. whether they have experience of being a patient, carer or healthcare professional) through a brief questionnaire distributed via email. Within the questionnaire, participants will be asked if they have already, or plan in the future, to complete ACP (see supplementary appendix).

Evaluation
Participants will have an opportunity to debrief and feedback on the day of the workshop. They will also have email contact details if they wish to provide written feedback between or after the sessions.

Follow-up
Following completion of the one-day workshop, participants will be contacted once approximately 4 weeks after the AEBCD event, via telephone or email to ask whether they have or plan to complete an ACP in the future. This follow-up phone call would last a maximum of 5 minutes and does not involve creating a FCP.

Study duration
We plan to conduct a minimum of one and a maximum of two AEBCD workshops. Each online workshop lasts the course of one day and is split into two separate half-day sessions. Completion of the one-day workshop should take approximately between 4 to 5 hours, including comfort breaks. Each participant would participate in only one workshop. The start date of the study is late summer 2021.

Data analysis
Responses from the participant questionnaire will be analysed to provide a descriptive summary of the participant characteristics. The information provided in Phase 1 (the discovery phase) will inform the co-design activities of session 2. On completion of the

For peer review only

workshop, the audio recordings will be transcribed verbatim. The principal investigator will listen to the recordings and review the transcripts. Other researchers will only have access to the transcripts. The ideas generated from the co-design workshop will be mapped onto the relevant components of the BCW and the behaviour change technique taxonomy (BCTT)(33); this will form the final content of the educational intervention. This content will be used to generate a prototype, which can be tested in future studies. The co-design process will be reported following guidance for reporting intervention development studies (34).

Data management and monitoring

The Principal Investigator will preserve the confidentiality of participants taking part in the study and fulfil transparency requirements under the General Data Protection Regulation for health and care research. Data and all appropriate documentation will be confidentially and securely stored for a minimum of 10 years after the completion of the study, including the follow-up period.

Consideration of well-being of participants

To respect participants' mental well-being, participants will be informed prior to the workshop that anyone can take a break whenever needed. Participants will be invited to speak with a mental health first aider after the meeting should the workshop raises any emotional concerns. To help participants feel at ease from the outset and reduce any power dynamics between professional clinicians and lay persons, there will be an informal icebreaker at the outset of the workshop. The facilitator will also highlight the value of both personal and professional experts throughout the workshop. During the workshop, participants will have access to a Jamboard (a virtual whiteboard via link in the chat function of Microsoft Teams). This will give attendees the option of writing personal/anonymous post-it notes to ensure that their comments are being captured.

Minimising digital exclusion

To reduce the risk of digital exclusion, participants will have an opportunity to read information on the software before the sessions to feel more comfortable. Participants will also be able to access any software before the start of the session and ask questions of the facilitator. During the workshop, participants can write comments within Microsoft Teams sessions for any further IT support.

Patient and public involvement:

This is a participatory action approach study. The design and delivery of the qualitative research presented in the discovery phase of the workshop involved older hospitalised patients, carers and healthcare professionals across medicine and surgery. The views and experiences of the public were also consulted in preparation for this study. Local primary care practitioners have provided feedback at an early stage on the design of the current study to ensure its relevance across different care settings. The concept behind the study, i.e. designing an intervention to increase uptake of advance care planning, has been discussed at an interdisciplinary stakeholder group with a specialist interest in advance care planning, including nursing leads and patient representatives.

For peer review only

Dissemination

In line with the final stage of the AEBCD method, the co-designed content will be “celebrated” and showcased in patient, carer and clinician forums, initially within Imperial College, the host organisation. The findings from this study will also be disseminated through academic publications, conferences and meetings

Authors’ contributions: AMB conceptualised and designed the study protocol under supervision from SA and CEU. AMB wrote the initial protocol. SA and CEU amended the initial protocol. All authors read, advised and approved of the protocol prior to submission.

Funding statement: The Department of Surgery and Cancer receives infrastructural support by the Imperial NIHR Biomedical Research Centre (BRC). This research received no specific grant to date from any funding agency in the public, commercial or not-for-profit sectors.

Competing interests statement: None declared

Acknowledgements: Anna Lawrence-Jones, Patient & Public Involvement & Engagement Lead at the Institute of Global Health Innovation, Imperial College London, for advice on facilitating online workshops to encourage collaboration within the co-design participant team.

For peer review only

Figure legends (see separate PDF files for figures)

Figure 1 – the Behaviour Change Wheel (reproduced from Michie et al, 2011)(20)

Figure 2. The steps of EBCD (adapted from Locock, 2014) (28)

Figure 3 Behaviour Change Wheel Steps for Intervention Development (adapted from Hall, 2020(22))

For peer review only

Reference list

1. Bielinska A, Archer S, Obanobi A, Soosaipillai G, Darzi A, Riley J, et al. Advance care planning in older hospitalised patients following an emergency admission: A mixed methods study. PLoS One. 2021;16((3):e0247874.).
2. Sudore RL, Lum HD, You JJ, Hanson LC, Meier DE, Pantilat SZ, et al. Defining Advance Care Planning for Adults: A Consensus Definition From a Multidisciplinary Delphi Panel. J Pain Symptom Manage. 2017;53(5):821-32 e1.
3. Austin CA, Mohottige D, Sudore RL, Smith AK, Hanson LC. Tools to Promote Shared Decision Making in Serious Illness: A Systematic Review. JAMA Intern Med. 2015;175(7):1213-21.
4. Lum HD, Sudore RL, Bekelman DB. Advance care planning in the elderly. Med Clin North Am. 2015;99(2):391-403.
5. Taylor K. Transforming care at the end-of-life. Dying well matters.: Deloitte Centre for Health Solutions; 2014.
6. Darzi A. The Lord Darzi Review of Health and Care: Interim report, IPPR.; 2018.
7. Combes S, Nicholson CJ, Gillett K, Norton C. Implementing advance care planning with community-dwelling frail elders requires a system-wide approach: An integrative review applying a behaviour change model. Palliat Med. 2019;33(7):743-56.
8. Steel AJ, Owen LH. Advance care planning: the who, what, when, where and why. Br J Hosp Med (Lond). 2020;81(2):1-6.
9. Clark D, Armstrong M, Allan A, Graham F, Carnon A, Isles C. Imminence of death among hospital inpatients: Prevalent cohort study. Palliative medicine. 2014;28(6):474-9.
10. Moore E, Munoz-Arroyo R, Schofield L, Radley A, Clark D, Isles C. Death within 1 year among emergency medical admissions to Scottish hospitals: incident cohort study. BMJ Open. 2018;8(6):e021432.
11. Bielinska AM, Archer S, Soosaipillai G, Riley J, Darzi LA, Urch C. Views of advance care planning in caregivers of older hospitalised patients following an emergency admission: A qualitative study. J Health Psychol. 2020:1359105320926547.
12. Detering KM, Hancock AD, Reade MC, Silvester W. The impact of advance care planning on end of life care in elderly patients: randomised controlled trial. BMJ. 2010;340:c1345.
13. Framework TGS. The Gold Standards Framework 2020 [Available from: https://goldstandardsframework.org.uk/evidence.
14. Callender T, Riley J, Broadhurst H, Wiggins N, Shaw A, Archer S, et al. The Determinants of Dying Where We Choose: An Analysis of Coordinate My Care. Ann Intern Med. 2017;167(7):519-21.
15. Waller A, Sanson-Fisher R, Nair BRK, Evans T. Are older and seriously ill inpatients planning ahead for future medical care? BMC Geriatr. 2019;19(1):212.
16. Waller A, Turon H, Bryant J, Zucca A, Evans TJ, Sanson-Fisher R. Medical oncology outpatients' preferences and experiences with advanced care planning: a cross-sectional study. BMC Cancer. 2019;19(1):63.
17. PeolcPSP. Palliative and end of life care Priority Setting Partnership (PeolcPSP). Putting patients, carers and clinicians at the heart of palliative and end of life care research.: James Lind Alliance; 2015.
18. Fried TR, Redding CA, Robbins ML, Paiva A, O'Leary JR, Iannone L. Promoting advance care planning as health behavior change: development of scales to assess Decisional Balance, Medical and Religious Beliefs, and Processes of Change. Patient Educ Couns. 2012;86(1):25-32.

For peer review only

19. Craig P, Dieppe P, Macintyre S, Michie S, Nazareth I, Petticrew M. Developing and evaluating complex interventions: the new Medical Research Council guidance. Int J Nurs Stud. 2013;50(5):587-92.
20. Michie S, van Stralen MM, West R. The behaviour change wheel: a new method for characterising and designing behaviour change interventions. Implement Sci. 2011;6:42.
21. PHE PHE-. Achieving Behaviour Change. A guide for national government. 2020.
22. Hall J, Morton S, Hall J, Clarke DJ, Fitzsimons CF, English C, et al. A co-production approach guided by the behaviour change wheel to develop an intervention for reducing sedentary behaviour after stroke. Pilot Feasibility Stud. 2020;6:115.
23. McAllister S, Simpson A, Tsianakas V, Canham N, De Meo V, Stone C, et al. Developing a theory-informed complex intervention to improve nurse-patient therapeutic engagement employing Experience-based Co-design and the Behaviour Change Wheel: an acute mental health ward case study. BMJ Open. 2021;11(5):e047114.
24. Kwok BC, Wong WP, Remedios L. Improving centre-based group exercise participation of older adults using the behaviour change wheel. BMJ Open Qual. 2021;10(1).
25. Loft MI, Martinsen B, Esbensen BA, Mathiesen LL, Iversen HK, Poulsen I. Strengthening the role and functions of nursing staff in inpatient stroke rehabilitation: developing a complex intervention using the Behaviour Change Wheel. Int J Qual Stud Health Well-being. 2017;12(sup2):1392218.
26. Ng BJ, Le Couteur DG, Hilmer SN. Deprescribing Benzodiazepines in Older Patients: Impact of Interventions Targeting Physicians, Pharmacists, and Patients. Drugs Aging. 2018;35(6):493-521.
27. Lavallee JF, Gray TA, Dumville JC, Cullum N. Preventing pressure injury in nursing homes: developing a care bundle using the Behaviour Change Wheel. BMJ Open. 2019;9(6):e026639.
28. Locock L, Robert G, Boaz A, Vougioukalou S, Shuldham C, Fielden J, et al. Using a national archive of patient experience narratives to promote local patient-centered quality improvement: an ethnographic process evaluation of 'accelerated' experience-based co-design. J Health Serv Res Policy. 2014;19(4):200-7.
29. Donetto S, Tsianakas, V. & Robert, G. Using Experience-based Co-design to improve the quality of healthcare: mapping where we are now and establishing future directions. London.: King’s College London.; 2014.
30. Bielinska A AS, Urch C, Darzi A Exploring healthcare professionals’ attitudes to future care planning with older adults in hospital: qualitative research co-design through multi-professional patient and carer involvement. BMJ Supportive & Palliative Care 2018;8(362).
31. Combes S, Gillett K, Norton C, Nicholson CJ. The importance of living well now and relationships: A qualitative study of the barriers and enablers to engaging frail elders with advance care planning. Palliat Med. 2021;35(6):1137-47.
32. Denvir MA, Murray SA, Boyd KJ. Future care planning: a first step to palliative care for all patients with advanced heart disease. Heart. 2015;101(13):1002-7.
33. Michie S RM, Johnston M, Abraham C, Francis J, Hardeman W, Eccles MP, Cane J, Wood CE. The behavior change technique taxonomy (v1) of 93 hierarchically clustered techniques: building an international consensus for the reporting of behavior change interventions. Ann Behav Med. 2013 46(1):81-95.
34. Duncan E, O'Cathain A, Rousseau N, Croot L, Sworn K, Turner KM, et al. Guidance for reporting intervention development studies in health research (GUIDED): an evidence-based consensus study. BMJ Open. 2020;10(4):e033516.

For peer review only

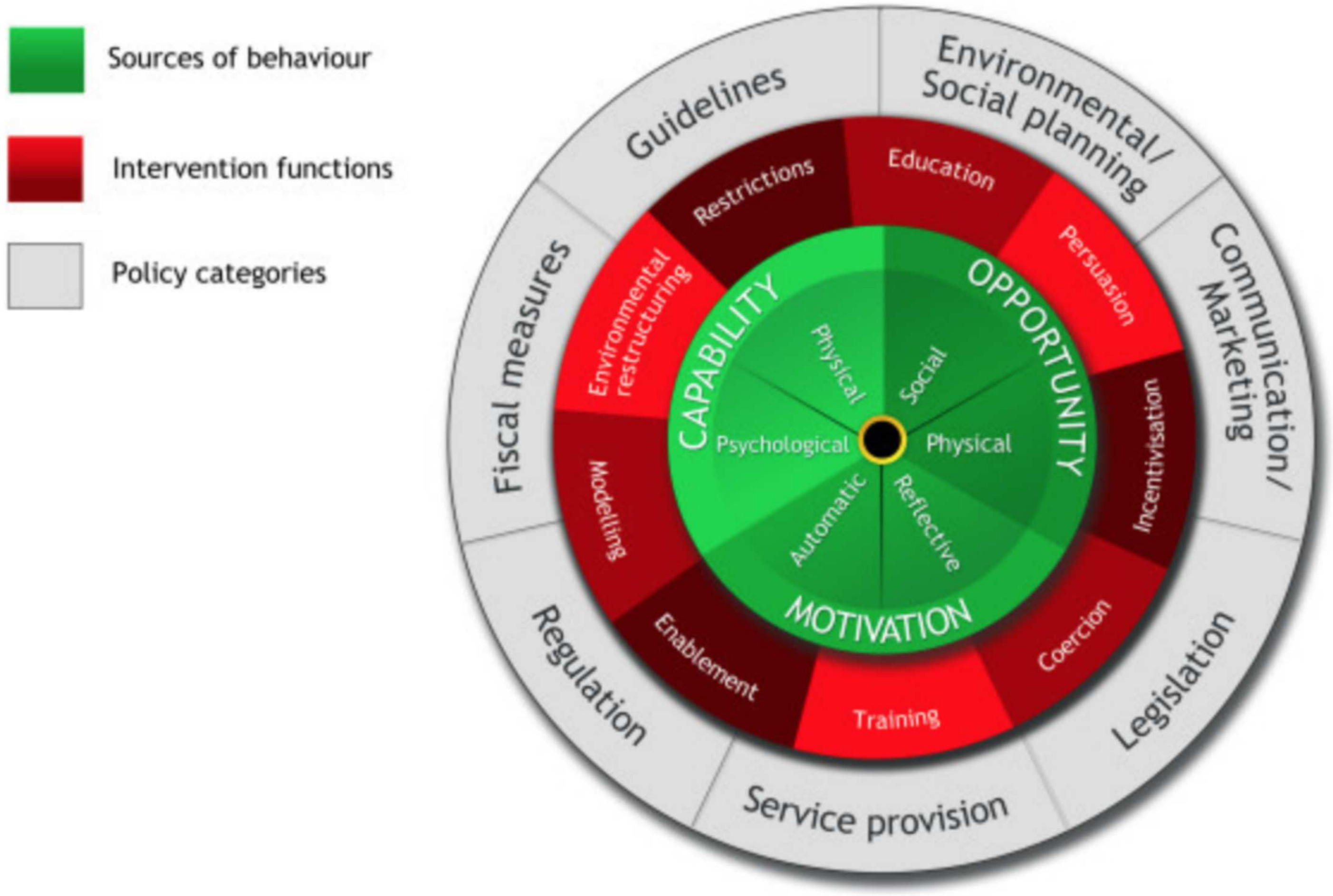
Sources of behaviour
Intervention functions
Policy categories
Guidelines
Environmental/
Social planning
Communication/
Marketing
Legislation
Service provision
Regulation
Fiscal measures
Restrictions
Education
Persuasion
Incentivisation
Coercion
Training
Enablement
Modelling
Environmental
restructuring
CAPABILITY
OPPORTUNITY
MOTIVATION
Physical
Psychological
Social
Physical
Automatic
Reflective

Figure 2. The steps of EBCD (adapted from Locock, 2014) (28)

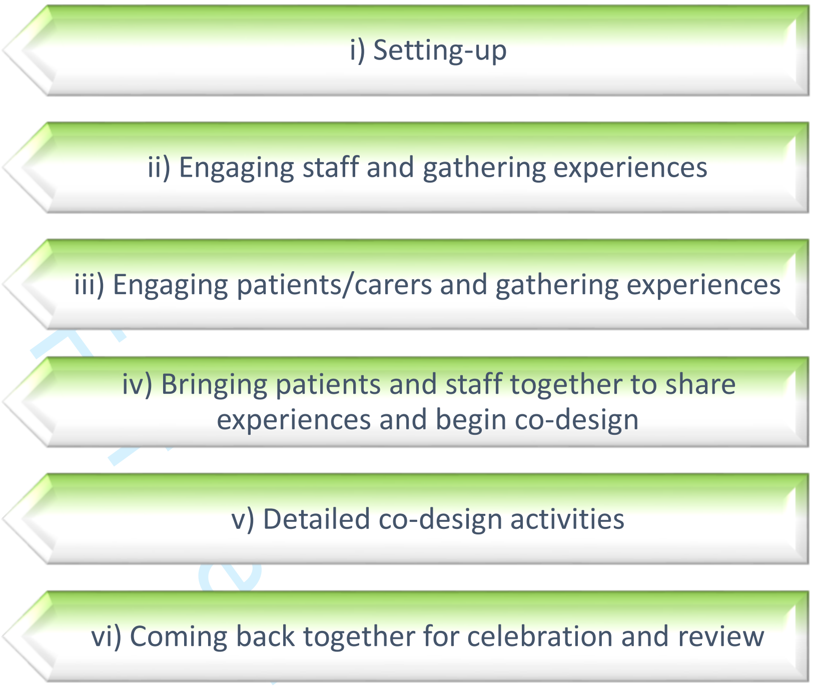

For peer review only

Figure 3 Behaviour Change Wheel Steps for Intervention Development (adapted from Hall, 2020(22))

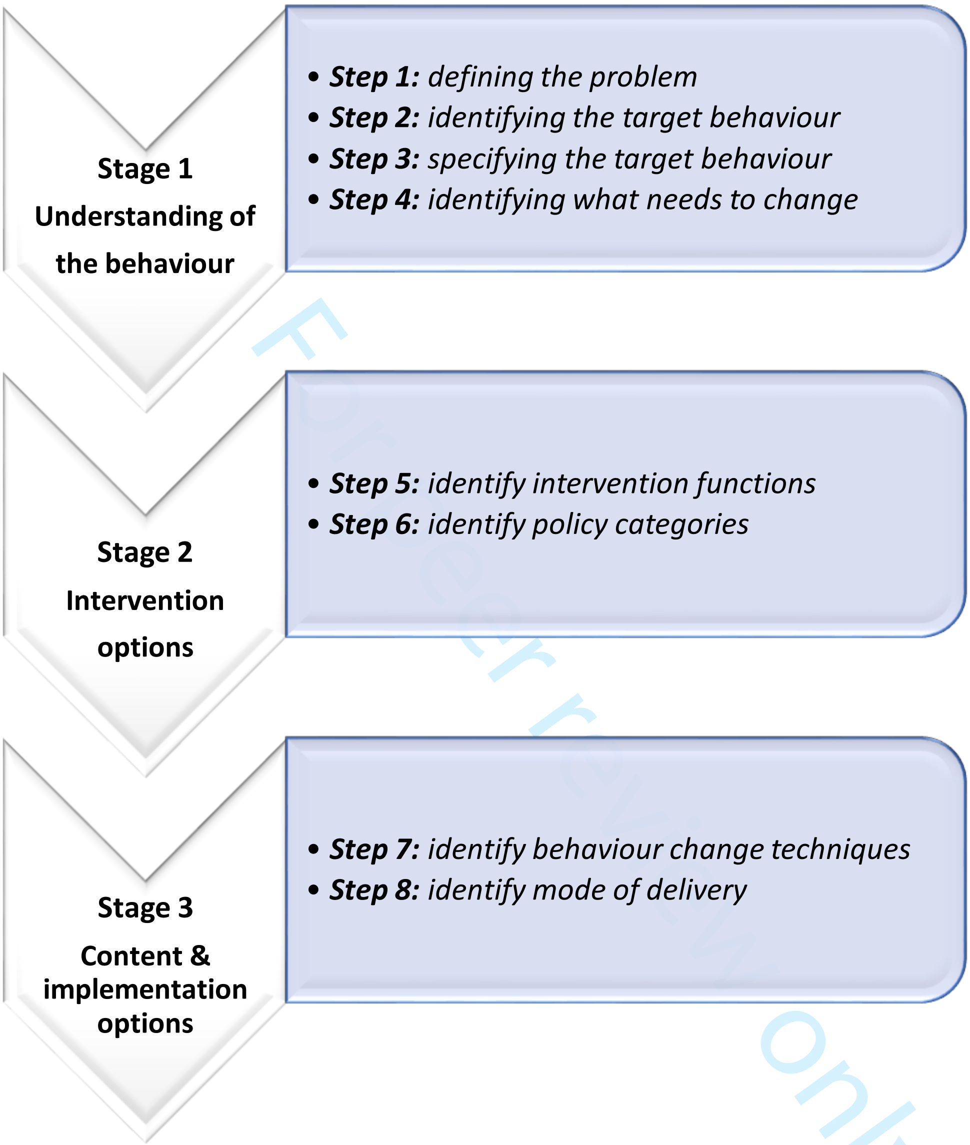

For peer review only

Supplementary appendix

Co-designing an intervention to increase uptake of Future Care Planning (FCP) in later life: Accelerated experience-based co-design (AEBCD) online workshop outline

Prior to online workshop

- Participants register beforehand by accepting Microsoft Teams invites (via email) and returning consent form (via e-mail).

- Participants complete brief questionnaire about their background & readiness towards FCP via email.

- Participants informed via email that anyone can take a break whenever they need and if the workshop raises any emotional concerns in people, they are welcome to speak to a mental health first aider after the meeting

- Participants encouraged to log-in early to workshop if possible (up to 15 mins beforehand if desired to check for any technical issues e.g. WiFi connectivity)

Resources needed for workshop sessions

- Internet access
- Microsoft Teams software (including breakout rooms, electronic chat on Teams)
- PowerPoint (using with share screen function on Microsoft Teams)
- Software for interactive answers, prioritisation and voting e.g. Mentimeter or equivalent
- Notepad to take additional field notes or use of virtual white board e.g. Jamboard
- Feedback forms

Persons involved in both sessions

- Principal investigator
- Co-investigator(s)
- Participants: 12 in total (consisting of 4 older persons, 4 carers and 4 healthcare professionals). An extra healthcare professional may need be invited in case of dropouts on the day of the workshop to balance the number of participants.

For peer review only

Timetable A: Half-day session 1 of 2 -DISCOVERY PHASE (Duration ~ 2 hours, 25 mins)

Activity	Description	Format	Deliverables	Time
Welcome & introduction	• Aims of workshop & brief agenda *(10mins)*<br>• Recap that both experts in experience and clinicians are participating in the workshop<br>• Information given to participants that anyone can take a break whenever needed and if the workshop raises any emotional issues, they are welcome to speak to a mental health first aider<br>• Icebreaker e.g. names, book/TV show/hobby enjoyed in lockdown and brief explanation of motivation to take part in the workshop *(5mins)*	Microsoft Teams	Responses on motivations to take part	~15mins
Discovery phase	• Overview of what Future Care Planning (FCP) is *(5mins)*<br>• Investigator(s) sharing results of previous projects exploring views of FCP in older adults of our different stakeholder groups (patients, carers, general public and healthcare professionals) *(20mins)*<br>• Reflection and discussion of different experiences, views and what informs FCP in later life, including amongst different stakeholder groups *(conducted in 2 smaller groups consisting of 2 older persons, 2 carers and 2 healthcare professionals, who can verbalise and use chat function of Teams, followed by a discussion in the main room with a 2-minute summary by each facilitator of the discussion) (25mins)*<br>*Comfort break (10 mins)*<br>• Discussion (*in breakout rooms with feedback to full group)*: Quick recap on FCP beforehand. Warm up question: Have you had experience in FCP? Why is FCP not more widely taken up? Discussion of FCP as a behaviour & barriers & facilitators *(20 mins)*<br>• Introduction to Behaviour Change Wheel (behaviour theory) *(5mins)*	PowerPoint<br>Guided Q & A<br>Open discussion<br>Nominal group technique<br>Small group & large group idea generation<br>Mentimeter poll	Responses from participants for<br>1) experiences & views on FCP<br>2) barriers & facilitators of FCP	~1 hour 30 mins

For peer review only

Preparation for co-design phase	• What would be useful to end-users to co-design? – reflection of useful initiatives and discussion/voting/decision. Use assessment to the process of developing, selecting and implementing behavioural interventions using the acronym APEASE **A**cceptability, **P**racticability, **E**ffectiveness, **A**ffordability, **S**ide-effects and **E**quity - (Public Health England, 2020) *(25mins)*<br><br>• Summarise concept for co-design ahead of session 2 *(5mins)*	PowerPoint<br><br>Q & A<br><br>Open discussion<br><br>Nominal Group Technique<br><br>Mentimeter poll	Responses and decision from participants for what to co-design	~30 mins
Conclusion & summary	• Conclusion/summary for first session<br><br>Next steps:<br>• Opportunity for feedback -contact details given for e-mail feedback if wished and mental health first aider details if the workshop has raised any difficult emotions for any participant.<br><br>• Encouragement to informally reflect on design between sessions (optional)<br><br>• Payment for participants (done via e-mail if wished)	PowerPoint	Feedback comments	~10 mins

For peer review only

Timetable B Half-day session 2 of 2 -CO-DESIGN PHASE USING THE BEHAVIOUR CHANGE WHEEL (Duration ~ 2 hours, 30 mins)

Activity	Description	Format	Deliverables	Time
Welcome back & recap	• Welcome back & recap to the workshop *(5mins)*<br>• Inform participants that anyone can take a break whenever they need and if the workshop raises any emotions, they are welcome to speak to a mental health first aider after the meeting<br>• Ice-breaker -re-introduce names *(5 mins)*<br>• Any comments/questions *(5mins)*	Microsoft Teams<br>PowerPoint		~15 mins
Co-design phase	• Recap on Behaviour Change Wheel and the process of developing, selecting, and implementing behavioural interventions using the acronym APEASE to drive the discussion - **A**cceptability, **P**racticability, **E**ffectiveness, **A**ffordability, **S**ide-effects and **E**quity -(Public Health England, 2020). *(10 mins)*<br>• Questions regarding the APEASE acronym (5 mins during/after discussing the APEASE criteria)<br>• Prioritising important values and behaviours for FCP in later life to different stakeholder groups in relation to Behaviour Change Wheel (patients, carers, clinicians) *(20 mins)*<br>• Co-design activity for an intervention to increase uptake FCP in older adults to guide social contacts and professionals using the Behavioural Change Wheel Method -consider the context/setting, format *(25 mins)*<br>*Comfort break 10 mins*<br>• Co-design activity continued, focusing on content (including detail) and linguistic approach of the intervention. *(25 mins)*<br>• Summary of intervention -overview, review using APEASE criteria, refinement and reflection on co-design activity *(20mins)*<br>• Discuss how this might be applied to policy *(10 mins)*	Guided Q & A<br>Open discussion<br>Nominal group technique<br>Ideas discussed in small groups with later feedback to larger groups<br>Selected Mentimeter poll	Responses on values & behaviours<br>Co-designed product - information on context, format, content and linguistic approach<br>APEASE criteria feedback<br>Ideas on policy applications	~2 hours

For peer review only

Conclusion	• Summary and thanks<br>• Opportunity for feedback, including details for mental health first aider in case the workshop has raised any difficult emotions<br>• Participants invited for voluntary follow-up call or email 4 weeks after the study to check readiness towards FCP<br>• Inform participants regarding forthcoming optional update about results/output of the study if participants agree			~15 mins

For peer review only

Co-designing an intervention to facilitate future care planning behaviours in later life

Participant questionnaire

What is your age? _________

1) **What is your gender?** *(Please tick)*

- ☐ *Male*
- ☐ *Female*
- ☐ *Other (please specify)* ______________________________
- ☐ *Prefer not to say*

2) **Please let us know about your background** *(please tick all that apply)*

- ☐ I have experience of being a patient
- ☐ I have experience of being a carer for an older person (aged 50+)
- ☐ I am a healthcare professional

If you are a healthcare professional, please tell us about your role

Type of healthcare professional (please specify e.g. nurse)

____

Specialty (please specify) ____________________________

Seniority (please specify) ____________________________

Years working in healthcare (please specify) ___________

Please feel free to tell us more about yourself in the space below
(e.g. your own experience of being a patient, caring or healthcare work, including any experience with emergency admissions to hospital).

For peer review only

3) How do you feel about future care planning for yourself? *(Please tick which statement best describes you)*

- *I have no need to plan my future care and I am not interested in this for myself whatsoever.*
- *I am aware future care planning might be relevant, but I am not planning to make a future care plan at this stage.*
- *I am planning on finding out more about future care planning and preparing to take the first steps to make a future care plan.*
- *I am in the process of making a future care plan for myself.*
- *I have made a future care plan for myself.*
- *I was interested in future care planning and/or had a future care plan but since then have changed my mind.*

If you are happy to be contacted 1 month after the study to be asked if you feel the same way about future care planning, please write your preferred contact details *(e.g. email/phone number).*

For peer review only

BMJ Open

Co-designing an intervention to increase uptake of Advance Care Planning in later life following emergency hospitalisation: a research protocol using Accelerated Experience-Based Co-design (AEBCD) and The Behaviour Change Wheel (BCW).

Journal:	*BMJ Open*
Manuscript ID	bmjopen-2021-055347.R1
Article Type:	Protocol
Date Submitted by the Author:	11-Jan-2022
Complete List of Authors:	Bielinska, Anna-Maria; Imperial College London, Department of Surgery and Cancer; Imperial College Healthcare NHS Trust<br>Archer, Stephanie; Imperial College London, Department of Surgery and Cancer; University of Cambridge, Department of Public Health and Primary Care and Department of Psychology<br>Darzi, Ara; Imperial College London, Department of Surgery and Cancer; Imperial College Healthcare NHS Trust<br>Urch, Catherine; Imperial College London Department of Surgery and Cancer; Imperial College Healthcare NHS Trust
**Primary Subject Heading**:	Health services research
Secondary Subject Heading:	Emergency medicine, Geriatric medicine, Palliative care, Medical education and training, Patient-centred medicine
Keywords:	GERIATRIC MEDICINE, PALLIATIVE CARE, ACCIDENT & EMERGENCY MEDICINE, QUALITATIVE RESEARCH, EDUCATION & TRAINING (see Medical Education & Training)

SCHOLARONE™
Manuscripts

TITLE PAGE

TITLE

Co-designing an intervention to increase uptake of Advance Care Planning in later life following emergency hospitalisation: a research protocol using Accelerated Experience-Based Co-design (AEBCD) and The Behaviour Change Wheel (BCW).

Lead & corresponding author

Dr Anna-Maria Bielinska[1,2], Clinical Research Fellow, Imperial College
Email: anna-maria.bielinska@doctors.org.uk
Postal address: Academic Surgical Unit, Department of Surgery and Cancer, 10th Floor QEQM Building, St Mary's Hospital, Praed Street, Paddington, London, W2 1NY

Co-authors:

Dr Stephanie Archer[1,3]
Professor Lord Ara Darzi[1,2]
Professor Catherine Urch[1,2]

Affiliations:

1) Department of Surgery and Cancer, Imperial College, London
2) Imperial College Healthcare NHS Trust, London
3) The University of Cambridge, UK

Word count 3987 words, excluding license statement, supplementary material and reference list

Planned study
Dates of study included in manuscript and initial cover letter

Ethical approval
obtained

For peer review only

License statement

I, the Submitting Author has the right to grant and does grant on behalf of all authors of the Work (as defined in the below author licence), an exclusive licence and/or a non-exclusive licence for contributions from authors who are: i) UK Crown employees; ii) where BMJ has agreed a CC-BY licence shall apply, and/or iii) in accordance with the terms applicable for US Federal Government officers or employees acting as part of their official duties; on a worldwide, perpetual, irrevocable, royalty-free basis to BMJ Publishing Group Ltd ("BMJ") its licensees and where the relevant Journal is co-owned by BMJ to the co-owners of the Journal, to publish the Work in BMJ Open and any other BMJ products and to exploit all rights, as set out in our licence.

The Submitting Author accepts and understands that any supply made under these terms is made by BMJ to the Submitting Author unless you are acting as an employee on behalf of your employer or a postgraduate student of an affiliated institution which is paying any applicable article publishing charge ("APC") for Open Access articles. Where the Submitting Author wishes to make the Work available on an Open Access basis (and intends to pay the relevant APC), the terms of reuse of such Open Access shall be governed by a Creative Commons licence – details of these licences and which Creative Commons licence will apply to this Work are set out in our licence referred to above.

For peer review only

Title:

Co-designing an intervention to increase uptake of Advance Care Planning in later life following emergency hospitalisation: a research protocol using Accelerated Experience-Based Co-design (AEBCD) and The Behaviour Change Wheel (BCW).

Abstract:

Introduction: Despite the potential benefits of advance care planning, uptake in older adults is low. In general, there is a lack of guidance as to how to initiate advance care planning conversations and encourage individuals to take action in planning their future care, including after emergency hospitalisation. Participatory action research methods are harnessed in health services research to design interventions that are relevant to end-users and stakeholders. This study aims to involve older persons, carers and healthcare professionals in co-designing an intervention to increase uptake of advance care planning in later life, which can be used by social contacts and healthcare professionals, particularly in the context of a recent emergency hospitalisation.

Methods and analysis: The theory-driven participatory design research method integrates and adapts Accelerated Experience-Based Co-Design with the Behaviour Change Wheel, in the form of a collaborative multi-stakeholder co-design workshop. In total, 12 participants, comprising 4 lay persons aged 70+, 4 carers and 4 healthcare professionals with experience in elder care, will be recruited to participate in two online half-day sessions, together comprising one online workshop. There will be a maximum of 2 workshops. Firstly, in the discovery phase, participants will reflect on findings from earlier qualitative research on views and experiences of advance care planning from three workstreams: patients, carers, and healthcare professionals. Secondly, in the co-design phase, participants will explore practical mechanisms in which older persons aged 70+ can be encouraged to adopt advance care planning behaviours based on the Behaviour Change Wheel, in order to co-design a behavioural intervention to increase uptake of advance care planning in older adults after an emergency hospitalisation.

Ethics and dissemination: Ethical approval has been obtained from the Science Engineering Technology Research Ethics Committee at Imperial College London (Reference:19IC5538). The findings from this study will be disseminated through publications, conferences and meetings.

For peer review only

Strengths and limitations of this study

1) The study draws upon previous research exploring the views of patients, carers and healthcare professionals in order to design a behavioural intervention.

2) Participants can reflect their own views and experiences during a highly collaborative co-design process, which recruits a diverse group of participants, including lay persons aged 70+, carers and healthcare professionals with experience in elder care.

3) To our knowledge this the first study to use the Behaviour Change Wheel to design a behavioural intervention to increase uptake of advance care planning in later life following an emergency hospitalisation.

4) To our knowledge this is also the first study combining Accelerated Experience Based Co-design with the BCW, other studies having used Experience Based Co-design over longer time frames.

5) The online workshop might digitally exclude older adults with limited access to the internet.

For peer review only

Introduction

Advance care planning (ACP) is a spectrum of approaches to planning future care (1) which "supports adults at any age or stage of health in understanding and sharing their personal values, life goals, and preferences" (2). ACP can be started at any point in an individual's life course, including during chronic and serious illness (3). It is an iterative process involving discussions between an individual and their care provider(s) and may cover a range of domains, from advance statements regarding care preferences, to advance decisions to refuse treatment (ADRT). Individuals may also decide to nominate a surrogate decision-maker in case of future loss of capacity (4). ACP has been highlighted as critical to supporting shared decision making between patients and healthcare professionals (1) through maintaining the autonomy and self-determination of patients. Research in older adults supports early engagement in ACP and highlights its importance in current and future care, as this group may have multi-morbidities, complex psychosocial needs and face social isolation (5-7).

ACP occurs in a range of settings including the home, general practice, nursing homes, specialist hospital outpatient services and hospital wards (4). Traditionally, ACP was mainly pursued in palliative care (8), however there is increased recognition that older hospitalised adults, particularly those who have had an emergency hospitalisation, are an important cohort for ACP (1, 9, 10). As around one in five patients aged 70+ admitted to hospital as an emergency are in the last year of life (1), acute hospitalisation can act as a trigger for tailored ACP (1, 10). ACP following hospitalisation is a dynamic process: preferences for life-sustaining treatment may change over the course of an acute illness or convalescence(11), and the impact of uncertainty in later life on ACP is recognised by caregivers of acutely hospitalised older persons(12). Qualitative research has shown that hospitalised older adults believe that ACP has a beneficial role in planning for their physical and psychosocial aspects of health, and patients look towards the collaborative expertise of clinicians to discuss and enact ACP (1). Furthermore, older inpatients also regard ACP as having a role in contemplating the possibility of physical deterioration, death and dying when considering healthcare choices in later life (1). Carers of older hospitalised adults regard ACP as process which helps to support older individuals, particularly in terms of well-being, quality of life and independence (12).

Despite the potential benefits of ACP in older adults receiving emergency hospital care, such as improved concordance of care with patient's wishes, improved end-of-life care and better emotional well-being in relatives (13), and notwithstanding the numerous care planning initiatives (14) (15), the uptake of ACP is low (16, 17). This is even amongst cohorts of patients who are facing life-changing illness, such as older and seriously unwell medial inpatients (16), surgical trauma patients(18, 19), and those attending cancer clinics (17). As such, it is essential that the uptake of ACP is increased in all patient groups (20), particularly older adults (21). Several studies have focused on brief interventions delivered by clinicians and facilitators, empowering seriously ill older persons to recognise the importance of ACP in the emergency department (22-24), with improved engagement in ACP and electronic documentation of health care proxy forms after emergency department visits(23). Whilst these interventions are extremely valuable in the emergency department, interventions to increase ACP that transcend other areas of health and or social care (e.g. on the hospital ward or in the community) are also required.

For peer review only

Rationale for the current study

Since ACP can be defined as a health behaviour, behaviour change models can help guide the development of interventions to increase the uptake of ACP (21). Greater understanding of ACP as a health behaviour is important as criticism of ACP has highlighted the lack of consideration of the "complexity, emotion and interpersonal elements of real-time decision making", as limiting its uptake and impact(18). Previous research has often limited ACP to completion of documentation or a one-off discussion with a clinician(18). Brief interventions to increase ACP have been used with success in the emergency department setting by clinicians and ACP facilitators(22-24). In contrast, our study examines ACP as behaviour to be explored within both a clinical and social context, using the event of an emergency hospitalisation as a pivotal "teachable" event to reflect on care planning needs in the future. Co-design involving older persons, carers and clinicians addressing ACP behaviour post hospitalisation in this context is novel.

The Medical Research Council recommends that identifying and developing a theoretical understanding is integral to developing complex interventions, including understanding the process of change, rationale, expected impact and the mechanisms by which change is to be achieved (25). Whilst a wide range of behaviour change frameworks exist (26), the Behaviour Change Wheel (BCW) has been cited as a recommended resource when designing interventions (27). The BCW is an evidence-based framework, which consists of a "behavioural COM-B system" at the hub, encircled by "intervention functions" and thereafter surrounded by "policy categories" (26) -see figure 1.

The COM-B system is a framework that can be used to understand a behaviour and focuses on capability, opportunity and motivation (26, 27). There are nine intervention functions: education, persuasion, incentivisation, coercion, training, restriction, environmental restructuring, modelling and enablement (26). The policy categories at the rim of the wheel support the delivery of the intervention (26). The BCW has been used to guide the development of a range of interventions in both physical and mental health (28, 29). Specifically in older adults' healthcare, the BCW has been used in a variety of behavioural interventions, from improving exercise participation for older adults in the community (30), to strengthening the role of nurses in stroke rehabilitation services(31), altering medication prescribing practices for older adults (32) and preventing pressure ulcers in nursing homes (33). Behavioural research by Sudore et al. has used the Stages of Change model (34) to understand ACP as a health behaviour(35). However, to date there are no published studies using the BCW to increase uptake of ACP in later life in any setting.

Whilst the BCW may be an appropriate and effective theoretical model on which to base the 'active ingredients' of an intervention, other approaches for their design and delivery must be considered, to deliver an intervention that is acceptable to those who will use it and/or implement it in their services. One such method is Experience Based Co-design (EBCD), which has been evaluated as an effective strategy for quality improvement in healthcare services through "narrative-based, participatory action research" (PAR) with patients, families and staff (36). Recent robust theory driven approaches have combined EBCD and the BCW to guide complex intervention development co-design, involving service users and other stakeholders (29). Both EBCD and the BCW are complementary and have been integrated in qualitative research (29). The stepwise, reflective and pragmatic approaches of

For peer review only - http://bmjopen.bmj.com/site/about/guidelines.xhtml

both EBCD and BCW help to provide solutions to benefit end-users in quality improvement initiatives (26, 36). EBCD involves six sequential steps (see figure 2).

Whilst the original ECBD format can vary in the length of time required to complete the process, typically lasting 6-12 months and occasionally exceeding this (37), a more expedited version is available (36). In *accelerated* experience-based co-design (AEBCD), pre-existing collected narratives of experience from relevant stakeholders such as patients and staff form the initial data to start co-design whilst maintaining the "rigorous and effective patient-centred quality improvement approach" (36).

Summary and aims

There is increasing evidence that an emergency admission may herald the last year of life in a subset of older adults (1, 10); increased uptake of ACP may be beneficial in this group. To address this need, the aim of this study is to use the BCW to co-design a behavioural intervention to increase uptake of ACP in later life after an emergency hospitalisation, through AEBCD with older persons, carers and healthcare professionals.

Methods and analysis

Study design

The research study will use AEBCD within an online workshop (split into two sessions) to co-design of an intervention to increase the uptake of ACP in the over 70s. In line with AEBCD methodology (36), the qualitative data collected from earlier studies (see box 1) will be used as a starting point.

Box 1: Prior work informing the design of this study

In preparation for this study, the experiences and views of older patients, carers and healthcare professionals of ACP in later life had been collected via qualitative interviews (1, 12, 38). Across three qualitative interview studies, a total of 48 participants took part - 20 hospitalised older adults, 8 carers and 20 frontline healthcare professionals. Interviews with older adults aged 70+ admitted to hospital have shown that they believe that ACP is relevant to their care following an emergency hospitalisation across a spectrum of prognoses and that they look towards the expertise of healthcare professionals after a medical emergency to plan their future care collaboratively (1). Carers of older adults who have been hospitalised believe that ACP has a role in supporting individuals in later life and that enabling the ACP process is crucial through practical support mechanisms (12). Interviews with frontline healthcare professionals have shown that open conversations with patients regarding ACP are valued in guiding clinicians' decisions and that there is a role in educating the clinical workforce to better understand how to approach ACP (unpublished data).

The AEBCD workshop will be divided into two phases: an initial discovery phase and a second co-design phase. The discovery phase will present the previously collected data on ACP. Workshop participants will be asked to reflect and discuss any experiences and views of ACP. This phase of the workshop will allow the group to identify and develop potential improvements to increase uptake of ACP (see session 1, timetable A).

The co-design phase will draw on the steps for intervention design using the BCW ((28); See Figure 3).

Whilst the BCW highlights the range of potential mechanisms which could be form the theoretical basis of our intervention, data from the prior qualitative research studies and wider literature has highlighted that increasing motivation via education may be efficacious in increasing uptake of ACP (1, 12, 39). As such, this stage of the codesign workshop will focus on developing the context/setting, format and content of an educational resource. This will be achieved through prioritising important values and behaviours for ACP in later life, and will consider the **a**cceptability, **p**racticability, **e**ffectiveness, **a**ffordability, **s**ide-effects and **e**quity (APEASE)(27) of any intervention (see timetable B).

Patient and public involvement

This is a participatory action approach study. The design and delivery of the qualitative research presented in the discovery phase of the workshop involved older hospitalised patients, carers and clinicians across medicine and surgery. The views and experiences of the public were consulted in preparation for this study. Local primary care practitioners provided feedback at an early stage of the study design to ensure its relevance across different care settings. The study concept, i.e., designing an intervention to increase uptake of ACP, was discussed at an interdisciplinary stakeholder group with a specialist interest in ACP, including nursing and patient representatives.

Study setting

The study will be conducted online on Microsoft Teams hosted by Imperial College.

Study participants

The AEBCD workshop will comprise of approximately 12 participants as follows:

- Four lay persons with experience of being a patient aged 70+ (ideally who have experienced an emergency admission)
- Four lay persons with experience of being a carer of an older person (particularly for those aged 70+)
- Four health and social care professionals with experience of caring for older patients aged 70+

We have decided to limit the number of participants per workshop to encourage in-depth discussions during a fixed-time period and encourage inter-participant and participant-facilitator interactions for productive co-design activities. However, there is scope for the workshop to be repeated, consistent with experience-based co-design methodology, where multiple activities are conducted over time (36).

Sampling method

The study will be advertised to lay persons via the Imperial Research Partners Group, Patient and Public Involvement initiatives and third sector. Lay persons interested in participating can contact the study team for more information. Healthcare professionals will be invited from Imperial College and other academic institutions, including via email to existing contacts who have experience of caring for older persons. Those who wish to participate will be able to respond by email to the study team.

Co-design workshops may attract participants with a specific interest in the subject area. To help reduce the risk of recruitment bias, the online platform VOICE will be used to recruit lay persons. VOICE is a general platform for public involvement in research, rather than a specialist organisation for end-of-life research (40). Furthermore, financial re-imbursement will be provided for lay participants, in line with guidance for public involvement by the National Institute for Health Research (NIHR) INVOLVE(41).

Where possible, purposive sampling will be used to achieve a diverse group of older persons and carers, in terms of a range of lived experience of illness, caring and ACP, in addition to purposive sampling of multi-disciplinary healthcare professionals across hospital and community settings. The VOICE platform for public involvement gathers information on equality and diversity, to help better understand the backgrounds of those involved in research, as per the recommendation of the NIHR. Any limitations in the diversity or inclusiveness of the sample will be acknowledged.

Participant inclusion criteria

The participant inclusion criteria are as follows:

- Participants must be able and willing to participate in a group discussion.
- Participants must have experience of being a patient, caring or are a healthcare professional with experience of older person's healthcare.
- Lay persons with experience of being a patient must be aged 70-years+
- Carers and healthcare professionals must be aged 18-years+
- Participants must have capacity to give informed consent
- Participants must be able to speak, read and write in English

Participant consent

Consent to enter the study will be sought from each participant after a full explanation has been given, a participant information sheet offered, and time allowed for consideration (at least 24 hours). Signed participant consent will be obtained. The right of the participant to refuse to participate without giving reasons will be respected and all participants will be free to withdraw at any time. If participants have already taken part in the workshop, their contribution will not be withdrawn as it contributes to a wider discussion, but no extracts from their contributions within the discussions will be used any write-up or presentation.

For peer review only

Study procedures

Terminology used

Some researchers use the term "future care planning" instead of "advance care planning" as this is more intuitively understood (42). "Future care planning" will be used in this study with participants to aid the flow of the discussion.

Baseline questionnaire

Data will be individually gathered on basic demographic information such as age, sex and background (e.g. whether they have experience of being a patient, carer or healthcare professional) through a brief questionnaire distributed via email. Within the questionnaire, participants will be asked if they have already, or plan in the future, to complete ACP (see supplementary appendix).

Evaluation

Participants will have an opportunity to debrief and feedback on the day of the workshop. They will also have email contact details if they wish to provide written feedback between or after the sessions.

Follow-up

Following completion of the one-day workshop, participants will be contacted once approximately 4 weeks after the AEBCD event, via telephone or email to ask whether they have or plan to complete an ACP in the future. This follow-up phone call would last a maximum of 5 minutes and does not involve creating an ACP. After the study, participants will be given the opportunity to receive updates regarding the study via email.

Study duration

We plan to conduct a minimum of one and a maximum of two AEBCD workshops.

The workshop will be repeated if further data is required following the initial workshop to form an intervention or guidance to facilitate ACP post-hospitalisation from the perspective of all three stakeholders (older persons, carers and clinicians), or if there is a need to purposefully recruit additional participants to achieve a balance of older persons, ideally with varied lived experience, carers and multidisciplinary clinicians

Each online workshop lasts the course of one day and is split into two separate half-day sessions. Completion of the one-day workshop should take approximately between 4 to 5 hours, including comfort breaks. Each participant would participate in only one workshop. The start date of the study is late summer 2021.

Data analysis

Responses from the participant questionnaire will be analysed to provide a descriptive summary of the participant characteristics. The information provided in Phase 1 (the discovery phase) will inform the co-design activities of session 2. On completion of the workshop, the audio recordings will be transcribed verbatim. The principal investigator will listen to the recordings and review the transcripts. Other researchers will only have access to the transcripts.

For peer review only

An inductive approach using thematic analysis(43) will be adopted. This 'bottom-up' approach to data analysis will allow us to explore elements related to the design of the intervention whilst more fully capturing the experiences of our participants that are not directly linked to intervention development. As a result, we will be able to use data from the study to inform intervention development (via the BCW/COM-B framework/BCTT) (44) and provide valuable insights into lived experiences of ACP, which may be disseminated separately. The co-design process will be reported following guidance for reporting intervention development studies (45).

We will use the findings of this study to generate a prototype educational intervention, which can be further refined and tested in future studies with a diverse group of participants. Other than a general reflection, developing specific detailed health policies is beyond the scope and time-constraints of the workshop - this would require future collaboration with the wider research team and other stakeholders relevant to the setting(s) where the policy may be implemented.

For peer review only

Ethics and dissemination

Ethical approval

Ethical approval has been obtained from the Science Engineering Technology Research Ethics Committee (SETREC) at Imperial College London (SETREC Reference: 19IC5538).

Ethical considerations

Informed consent of participants is integral to the study as already outlined in the study methods. Ethical issues relating to data management, participant well-being and digital exclusion are detailed below.

Data management and monitoring

The Principal Investigator will preserve the confidentiality of participants taking part in the study and fulfil transparency requirements under the General Data Protection Regulation for health and care research. Data and all appropriate documentation will be confidentially and securely stored for a minimum of 10 years after the completion of the study, including the follow-up period.

Consideration of well-being of participants

To respect participants' mental well-being, participants will be informed prior to the workshop that anyone can take a break whenever needed. Participants will be invited to speak with a mental health first aider after the meeting should the workshop raises any emotional concerns. To help participants feel at ease from the outset and reduce any power dynamics between professional clinicians and lay persons, there will be an informal icebreaker at the outset of the workshop. The facilitator will also highlight the value of both personal and professional experts throughout the workshop. If necessary, participants may access Jamboard (a virtual whiteboard via link in the chat function of Microsoft Teams). This will give attendees the option of writing personal/anonymous post-it notes to ensure that their comments are being captured.

Minimising digital exclusion

To reduce the risk of digital exclusion, participants will have an opportunity to read software information before sessions, if needed, to feel more comfortable. Participants will also be able to access any software before the start of the session and ask questions. During the workshop, participants can write comments within Microsoft Teams sessions for any further IT support.

Dissemination

In line with the final stage of the AEBCD method, the co-designed content will be "celebrated" and showcased in patient, carer and clinician forums, initially within Imperial College, the host organisation. The findings from this study will also be disseminated through academic publications, conferences and meetings

For peer review only

Figure legends (see separate PDF files for figures)

Figure 1 – the Behaviour Change Wheel (reproduced from Michie et al, 2011)(26)

Figure 2. The steps of EBCD (adapted from Locock, 2014) (28)

Figure 3 Behaviour Change Wheel Steps for Intervention Development (adapted from Hall, 2020(28))

For peer review only

Acknowledgements: Anna Lawrence-Jones, Patient & Public Involvement & Engagement Lead at the Institute of Global Health Innovation, Imperial College London, for advice on facilitating online workshops to encourage collaboration within the co-design participant team. Soh-yon Park, Design Researcher at the Institute of Global Health Innovation, Imperial College London, for expertise on facilitating workshop discussions.

Contributorship statement: AMB conceptualised and designed the study protocol under supervision from SA and CEU. AMB wrote the initial protocol. SA and CEU amended the initial protocol. AMB, SA, AD and CEU read, advised and approved of the protocol prior to submission.

Competing interests: None declared

Funding: The Department of Surgery and Cancer receives infrastructural support by the Imperial NIHR Biomedical Research Centre (BRC). Funding granted via the National Institute for Health Research Patient Safety Translation Research Centre (NIHR PSTRC) - grant reference number PSTRC-2016-004

For peer review only

Reference list

1. Bielinska A, Archer S, Obanobi A, Soosaipillai G, Darzi A, Riley J, et al. Advance care planning in older hospitalised patients following an emergency admission: A mixed methods study. PLoS One. 2021;16((3):e0247874.).
2. Sudore RL, Lum HD, You JJ, Hanson LC, Meier DE, Pantilat SZ, et al. Defining Advance Care Planning for Adults: A Consensus Definition From a Multidisciplinary Delphi Panel. J Pain Symptom Manage. 2017;53(5):821-32 e1.
3. Austin CA, Mohottige D, Sudore RL, Smith AK, Hanson LC. Tools to Promote Shared Decision Making in Serious Illness: A Systematic Review. JAMA Intern Med. 2015;175(7):1213-21.
4. Lum HD, Sudore RL, Bekelman DB. Advance care planning in the elderly. Med Clin North Am. 2015;99(2):391-403.
5. Taylor K. Transforming care at the end-of-life. Dying well matters.: Deloitte Centre for Health Solutions; 2014.
6. Darzi A. The Lord Darzi Review of Health and Care: Interim report, IPPR.; 2018.
7. Combes S, Nicholson CJ, Gillett K, Norton C. Implementing advance care planning with community-dwelling frail elders requires a system-wide approach: An integrative review applying a behaviour change model. Palliat Med. 2019;33(7):743-56.
8. Steel AJ, Owen LH. Advance care planning: the who, what, when, where and why. Br J Hosp Med (Lond). 2020;81(2):1-6.
9. Clark D, Armstrong M, Allan A, Graham F, Carnon A, Isles C. Imminence of death among hospital inpatients: Prevalent cohort study. Palliative medicine. 2014;28(6):474-9.
10. Moore E, Munoz-Arroyo R, Schofield L, Radley A, Clark D, Isles C. Death within 1 year among emergency medical admissions to Scottish hospitals: incident cohort study. BMJ Open. 2018;8(6):e021432.
11. Ditto PH, Jacobson JA, Smucker WD, Danks JH, Fagerlin A. Context changes choices: a prospective study of the effects of hospitalization on life-sustaining treatment preferences. Med Decis Making. 2006;26(4):313-22.
12. Bielinska AM, Archer S, Soosaipillai G, Riley J, Darzi LA, Urch C. Views of advance care planning in caregivers of older hospitalised patients following an emergency admission: A qualitative study. J Health Psychol. 2020:1359105320926547.
13. Detering KM, Hancock AD, Reade MC, Silvester W. The impact of advance care planning on end of life care in elderly patients: randomised controlled trial. BMJ. 2010;340:c1345.
14. Framework TGS. The Gold Standards Framework 2020 [Available from: https://goldstandardsframework.org.uk/evidence.
15. Callender T, Riley J, Broadhurst H, Wiggins N, Shaw A, Archer S, et al. The Determinants of Dying Where We Choose: An Analysis of Coordinate My Care. Ann Intern Med. 2017;167(7):519-21.
16. Waller A, Sanson-Fisher R, Nair BRK, Evans T. Are older and seriously ill inpatients planning ahead for future medical care? BMC Geriatr. 2019;19(1):212.
17. Waller A, Turon H, Bryant J, Zucca A, Evans TJ, Sanson-Fisher R. Medical oncology outpatients' preferences and experiences with advanced care planning: a cross-sectional study. BMC Cancer. 2019;19(1):63.
18. Morrison RS. Advance Directives/Care Planning: Clear, Simple, and Wrong. J Palliat Med. 2020;23(7):878-9.

For peer review only

19. Yadav KN, Gabler NB, Cooney E, Kent S, Kim J, Herbst N, et al. Approximately One In Three US Adults Completes Any Type Of Advance Directive For End-Of-Life Care. Health Aff (Millwood). 2017;36(7):1244-51.
20. PeolcPSP. Palliative and end of life care Priority Setting Partnership (PeolcPSP). Putting patients, carers and clinicians at the heart of palliative and end of life care research.: James Lind Alliance; 2015.
21. Fried TR, Redding CA, Robbins ML, Paiva A, O'Leary JR, Iannone L. Promoting advance care planning as health behavior change: development of scales to assess Decisional Balance, Medical and Religious Beliefs, and Processes of Change. Patient Educ Couns. 2012;86(1):25-32.
22. Ouchi K, George N, Revette AC, Hasdianda MA, Fellion L, Reust A, et al. Empower Seriously Ill Older Adults to Formulate Their Goals for Medical Care in the Emergency Department. J Palliat Med. 2019;22(3):267-73.
23. Pajka SE, Hasdianda MA, George N, Sudore R, Schonberg MA, Bernstein E, et al. Feasibility of a Brief Intervention to Facilitate Advance Care Planning Conversations for Patients with Life-Limiting Illness in the Emergency Department. J Palliat Med. 2021;24(1):31-9.
24. Leiter RE, Yusufov M, Hasdianda MA, Fellion LA, Reust AC, Block SD, et al. Fidelity and Feasibility of a Brief Emergency Department Intervention to Empower Adults With Serious Illness to Initiate Advance Care Planning Conversations. J Pain Symptom Manage. 2018;56(6):878-85.
25. Craig P, Dieppe P, Macintyre S, Michie S, Nazareth I, Petticrew M. Developing and evaluating complex interventions: the new Medical Research Council guidance. Int J Nurs Stud. 2013;50(5):587-92.
26. Michie S, van Stralen MM, West R. The behaviour change wheel: a new method for characterising and designing behaviour change interventions. Implement Sci. 2011;6:42.
27. PHE PHE-. Achieving Behaviour Change. A guide for national government. 2020.
28. Hall J, Morton S, Hall J, Clarke DJ, Fitzsimons CF, English C, et al. A co-production approach guided by the behaviour change wheel to develop an intervention for reducing sedentary behaviour after stroke. Pilot Feasibility Stud. 2020;6:115.
29. McAllister S, Simpson A, Tsianakas V, Canham N, De Meo V, Stone C, et al. Developing a theory-informed complex intervention to improve nurse-patient therapeutic engagement employing Experience-based Co-design and the Behaviour Change Wheel: an acute mental health ward case study. BMJ Open. 2021;11(5):e047114.
30. Kwok BC, Wong WP, Remedios L. Improving centre-based group exercise participation of older adults using the behaviour change wheel. BMJ Open Qual. 2021;10(1).
31. Loft MI, Martinsen B, Esbensen BA, Mathiesen LL, Iversen HK, Poulsen I. Strengthening the role and functions of nursing staff in inpatient stroke rehabilitation: developing a complex intervention using the Behaviour Change Wheel. Int J Qual Stud Health Well-being. 2017;12(sup2):1392218.
32. Ng BJ, Le Couteur DG, Hilmer SN. Deprescribing Benzodiazepines in Older Patients: Impact of Interventions Targeting Physicians, Pharmacists, and Patients. Drugs Aging. 2018;35(6):493-521.
33. Lavallee JF, Gray TA, Dumville JC, Cullum N. Preventing pressure injury in nursing homes: developing a care bundle using the Behaviour Change Wheel. BMJ Open. 2019;9(6):e026639.
34. Prochaska JO, DiClemente CC. Stages and processes of self-change of smoking: toward an integrative model of change. J Consult Clin Psychol. 1983;51(3):390-5.

For peer review only

35. Sudore RL, Knight SJ, McMahan RD, Feuz M, Farrell D, Miao Y, et al. A novel website to prepare diverse older adults for decision making and advance care planning: a pilot study. J Pain Symptom Manage. 2014;47(4):674-86.
36. Locock L, Robert G, Boaz A, Vougioukalou S, Shuldham C, Fielden J, et al. Using a national archive of patient experience narratives to promote local patient-centered quality improvement: an ethnographic process evaluation of 'accelerated' experience-based co-design. J Health Serv Res Policy. 2014;19(4):200-7.
37. Donetto S, Tsianakas, V. & Robert, G. Using Experience-based Co-design to improve the quality of healthcare: mapping where we are now and establishing future directions. London.: King's College London.; 2014.
38. Bielinska A AS, Urch C, Darzi A Exploring healthcare professionals' attitudes to future care planning with older adults in hospital: qualitative research co-design through multi-professional patient and carer involvement. BMJ Supportive & Palliative Care 2018;8(362).
39. Combes S, Gillett K, Norton C, Nicholson CJ. The importance of living well now and relationships: A qualitative study of the barriers and enablers to engaging frail elders with advance care planning. Palliat Med. 2021;35(6):1137-47.
40. Voice. Voice: VOICE; 2022 [Available from: https://www.voice-global.org.
41. NIHR. Payment guidance for researchers and professionals: National Institute for Health Research; 2021 [updated 5.4.21. V1.0:[Available from: https://www.nihr.ac.uk/documents/payment-guidance-for-researchers-and-professionals/27392.
42. Denvir MA, Murray SA, Boyd KJ. Future care planning: a first step to palliative care for all patients with advanced heart disease. Heart. 2015;101(13):1002-7.
43. Braun VC, Victoria. Using Thematic Analysis in Psychology. Qualitative Research in Psychology. 2006;3(2):77-101.
44. Michie S RM, Johnston M, Abraham C, Francis J, Hardeman W, Eccles MP, Cane J, Wood CE. The behavior change technique taxonomy (v1) of 93 hierarchically clustered techniques: building an international consensus for the reporting of behavior change interventions. Ann Behav Med. 2013 46(1):81-95.
45. Duncan E, O'Cathain A, Rousseau N, Croot L, Sworn K, Turner KM, et al. Guidance for reporting intervention development studies in health research (GUIDED): an evidence-based consensus study. BMJ Open. 2020;10(4):e033516.

For peer review only

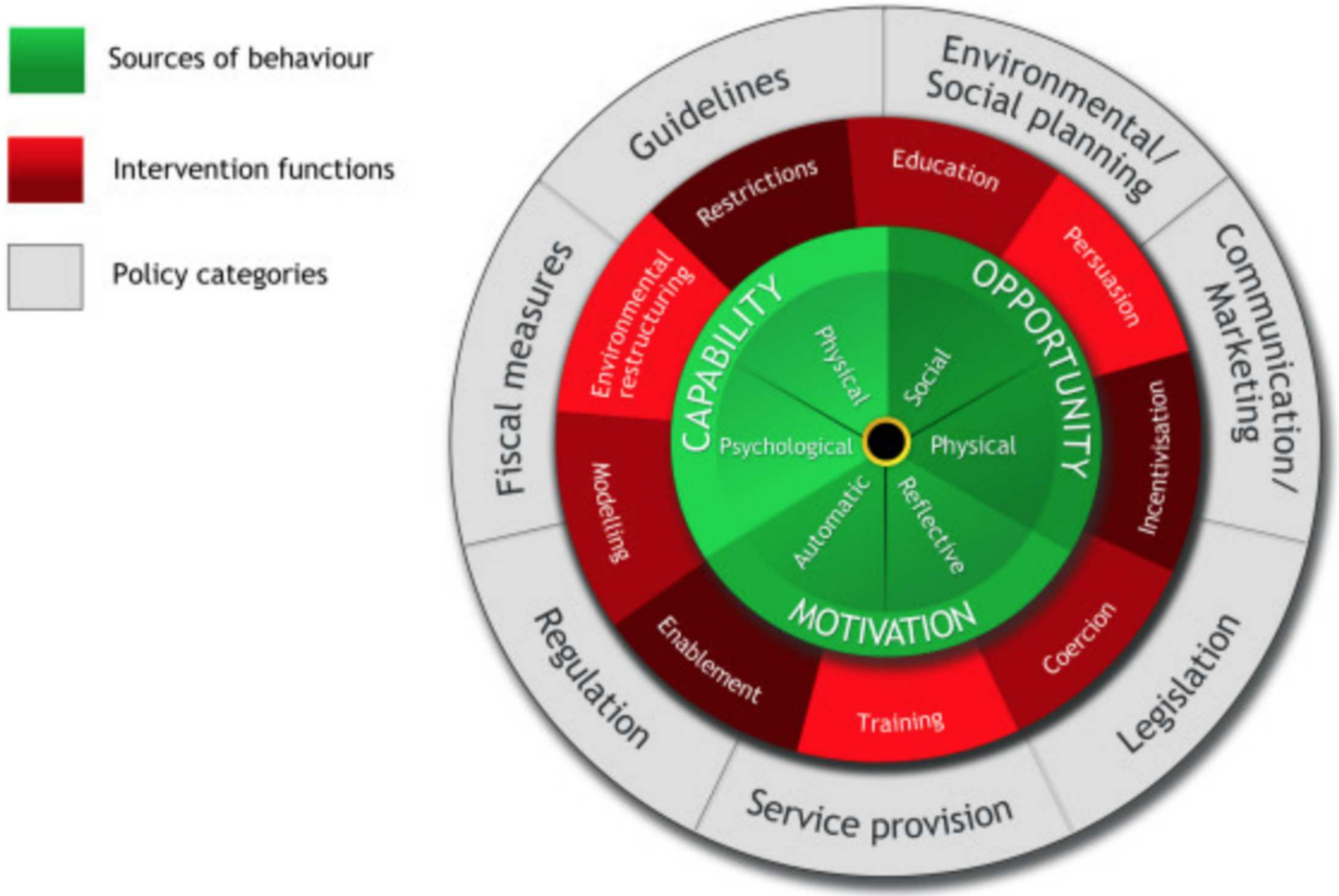
Sources of behaviour
Intervention functions
Policy categories
Guidelines
Environmental/
Social planning
Communication/
Marketing
Legislation
Service provision
Regulation
Fiscal measures
Restrictions
Education
Persuasion
Incentivisation
Coercion
Training
Enablement
Modelling
Environmental restructuring
CAPABILITY
OPPORTUNITY
MOTIVATION
Physical
Social
Psychological
Physical
Automatic
Reflective

Figure 2. The steps of EBCD (adapted from Locock, 2014) (28)

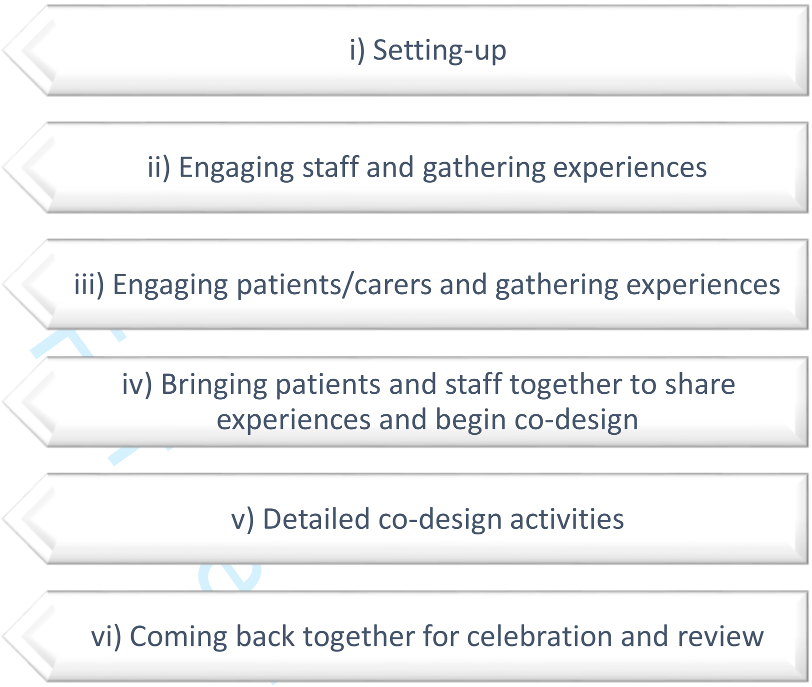

For peer review only

Figure 3 Behaviour Change Wheel Steps for Intervention Development (adapted from Hall, 2020(22))

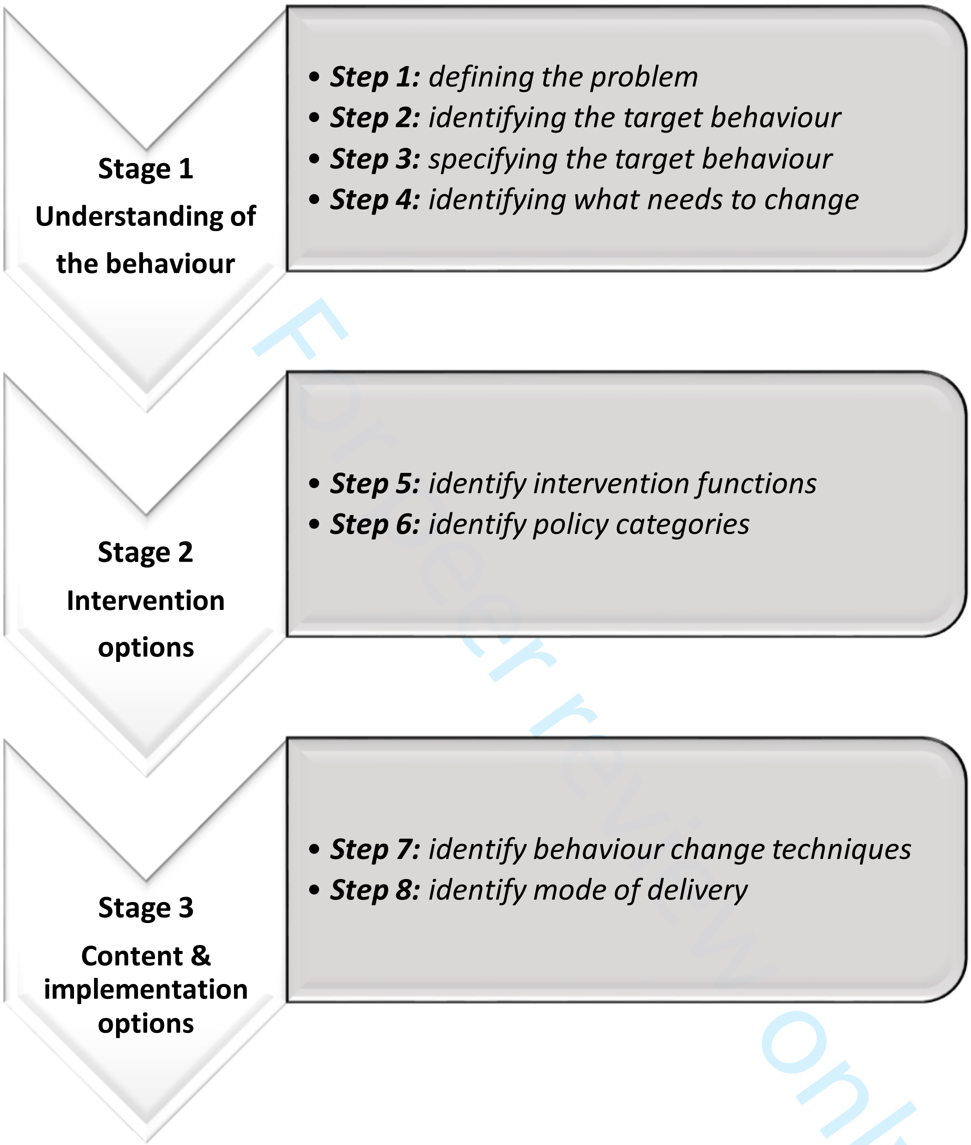

Supplementary appendix

Co-designing an intervention to increase uptake of Advance Care Planning (ACP) in later life: Accelerated experience-based co-design (AEBCD) online workshop outline

Prior to online workshop

- Participants register beforehand by accepting Microsoft Teams invites (via email) and returning consent form (via e-mail).
- Participants complete brief questionnaire about their background & readiness towards ACP via email.
- Participants informed via email that anyone can take a break whenever they need and if the workshop raises any emotional concerns in people, they are welcome to speak to a mental health first aider after the meeting
- Participants encouraged to log-in early to workshop if possible (up to 15 mins beforehand if desired to check for any technical issues e.g. WiFi connectivity)
- Recognition within the facilitator and co-design team that an element of flexibility in the schedule may be required on the day to respond to participants' needs and contributions during the live workshop.

Resources needed for workshop sessions

- Internet access
- Microsoft Teams software (including breakout rooms, electronic chat on Teams)
- PowerPoint (using with share screen function on Microsoft Teams)
- Software for interactive answers, prioritisation and voting e.g. Mentimeter or equivalent
- Notepad to take additional field notes or use of virtual white board e.g. Jamboard
- Feedback forms

Persons involved in both sessions

- Principal investigator (to act as main facilitator)
- Co-investigators (to act as assistant facilitators)
- Participants: 12 in total (consisting of 4 older persons, 4 carers and 4 healthcare professionals). An extra healthcare professional may need be invited in case of dropouts on the day of the workshop to balance the number of participants.

For peer review only

Role designation of workshop team to facilitate discussion

Role of the main facilitator

The discussion will be facilitated by a main facilitator presenting the content of previous research and asking participants questions about their experiences and views, with an emphasis on open questioning, asking probing questions where appropriate and clarifying participants' comments. The main facilitator will also answer any other verbal questions that participants may have during the workshop, particularly related to ACP and behaviour change.

Role of the assistant facilitators

In order to focus on presentation of content for the discovery phase and driving the verbal discussion during the workshop, the main facilitator will be assisted by two assistant facilitators for technical support, note-taking and timekeeping.

The first assistant facilitator will take notes, attend to any technical software questions, and field text group chat questions in the Microsoft Teams interface, with the support of the second facilitator on this latter task if necessary.

The second assistant facilitator will focus on time-keeping of the workshop, and ensuring all voices are heard and considered, including keeping track of which participants have spoken, which participants wish to further contribute, including noting the order of participants "raising hands" on Microsoft Teams.

For peer review only

Timetable A: Half-day session 1 of 2 -DISCOVERY PHASE (Duration ~ 2 hours, 25 mins)

Activity	Description	Format	Deliverables	Time
Welcome & introduction	• Aims of workshop & brief agenda *(10mins)*<br>• Recap that both experts in experience and clinicians are participating in the workshop<br>• Information given to participants that anyone can take a break whenever needed and if the workshop raises any emotional issues, they are welcome to speak to a mental health first aider<br>• Icebreaker e.g., names, book/TV show/hobby enjoyed in lockdown and brief explanation of motivation to take part in the workshop *(5mins)*	Microsoft Teams	Responses on motivations to take part	~15mins
Discovery phase	• Overview of what Advance Care Planning (ACP) is *(5mins)*<br>• Investigator(s) sharing results of previous projects exploring views of ACP in older adults of our different stakeholder groups (patients, carers, general public and healthcare professionals) *(20mins)*<br>• Reflection and discussion of different experiences, views and what informs ACP in later life, including amongst different stakeholder groups *(conducted in 2 smaller groups consisting of 2 older persons, 2 carers and 2 healthcare professionals, who can verbalise and use chat function of Teams, followed by a discussion in the main room with a 2-minute summary by each facilitator of the discussion) (25mins)*<br>*Comfort break (10 mins)*<br>• Discussion (*in breakout rooms with feedback to full group)*: Quick recap on ACP beforehand. Warm up question: Have you had experience in ACP? Why is ACP not more widely taken up? Discussion of ACP as a behaviour & barriers & facilitators *(20 mins)*<br>• Introduction to Behaviour Change Wheel (behaviour theory) *(5mins)*	PowerPoint<br>Guided Q & A<br>Open discussion<br>Nominal group technique<br>Small group & large group idea generation<br>Mentimeter poll	Responses from participants for<br>1) experiences & views on ACP<br>2) barriers & facilitators of ACP	~1 hour 30 mins

For peer review only

Preparation for co-design phase	• What would be useful to end-users to co-design? – reflection of useful initiatives and discussion/voting/decision. Use assessment to the process of developing, selecting and implementing behavioural interventions using the acronym APEASE **A**cceptability, **P**racticability, **E**ffectiveness, **A**ffordability, **S**ide-effects and **E**quity - (Public Health England, 2020) *(25mins)*<br><br>• Summarise concept for co-design ahead of session 2 *(5mins)*	PowerPoint<br><br>Q & A<br><br>Open discussion<br><br>Nominal Group Technique<br><br>Mentimeter poll	Responses and decision from participants for what to co-design	~30 mins
Conclusion & summary	• Conclusion/summary for first session<br><br>Next steps:<br>• Opportunity for feedback -contact details given for e-mail feedback if wished and mental health first aider details if the workshop has raised any difficult emotions for any participant.<br><br>• Encouragement to informally reflect on design between sessions (optional)<br><br>• Payment for participants (done via e-mail if wished)	PowerPoint	Feedback comments	~10 mins

For peer review only

Timetable B Half-day session 2 of 2 -CO-DESIGN PHASE USING THE BEHAVIOUR CHANGE WHEEL (Duration ~ 2 hours, 30 mins)

Activity	Description	Format	Deliverables	Time
Welcome back & recap	• Welcome back & recap to the workshop *(5mins)*<br>• Inform participants that anyone can take a break whenever they need and if the workshop raises any emotions, they are welcome to speak to a mental health first aider after the meeting<br><br>• Ice-breaker -re-introduce names *(5 mins)*<br><br>• Any comments/questions *(5mins)*	Microsoft Teams<br><br>PowerPoint		~15 mins
Co-design phase	• Recap on Behaviour Change Wheel and the process of developing, selecting, and implementing behavioural interventions using the acronym APEASE to drive the discussion - **A**cceptability, **P**racticability, **E**ffectiveness, **A**ffordability, **S**ide-effects and **E**quity -(Public Health England, 2020). *(10 mins)*<br><br>• Questions regarding the APEASE acronym (5 mins during/after discussing the APEASE criteria)<br><br>• Prioritising important values and behaviours for ACP in later life to different stakeholder groups in relation to Behaviour Change Wheel (patients, carers, clinicians) *(20 mins)*<br><br>• Co-design activity for an intervention to increase uptake ACP in older adults to guide social contacts and professionals using the Behavioural Change Wheel Method -consider the context/setting, format *(25 mins)*<br><br>*Comfort break 10 mins*<br><br>• Co-design activity continued, focusing on content (including detail) and linguistic approach of the intervention. *(25 mins)*<br><br>• Summary of intervention -overview, review using APEASE criteria, refinement and reflection on co-design activity *(20mins)*<br><br>• Discuss how this might be applied to policy *(10 mins)*	Guided Q & A<br><br>Open discussion<br><br>Nominal group technique<br><br>Ideas discussed in small groups with later feedback to larger groups<br><br>Selected Mentimeter poll	Responses on values & behaviours<br><br>Co-designed product - information on context, format, content and linguistic approach<br><br>APEASE criteria feedback<br><br>Ideas on policy applications	~2 hours

For peer review only

Conclusion	• Summary and thanks<br>• Opportunity for feedback, including details for mental health first aider in case the workshop has raised any difficult emotions<br>• Participants invited for voluntary follow-up call or email 4 weeks after the study to check readiness towards ACP<br>• Inform participants regarding forthcoming optional update about results/output of the study if participants agree			~15 mins

For peer review only

Co-designing an intervention to facilitate future care planning behaviours in later life

Participant questionnaire

What is your age? _________

1) **What is your gender?** *(Please tick)*
   - *Male*
   - *Female*
   - *Other (please specify)* ___________________________
   - *Prefer not to say*
2) **Please let us know about your background** *(please tick all that apply)*
   - I have experience of being a patient
   - I have experience of being a carer for an older person (aged 50+)
   - I am a healthcare professional

If you are a healthcare professional, please tell us about your role

Type of healthcare professional (please specify e.g. nurse)

____

Specialty (please specify) _________________________

Seniority (please specify) _________________________

Years working in healthcare (please specify) __________

Please feel free to tell us more about yourself in the space below
(e.g. your own experience of being a patient, caring or healthcare work, including any experience with emergency admissions to hospital).

For peer review only

3) How do you feel about future care planning for yourself? *(Please tick which statement best describes you)*

- *I have no need to plan my future care and I am not interested in this for myself whatsoever.*
- *I am aware future care planning might be relevant, but I am not planning to make a future care plan at this stage.*
- *I am planning on finding out more about future care planning and preparing to take the first steps to make a future care plan.*
- *I am in the process of making a future care plan for myself.*
- *I have made a future care plan for myself.*
- *I was interested in future care planning and/or had a future care plan but since then have changed my mind.*

If you are happy to be contacted 1 month after the study to be asked if you feel the same way about future care planning, please write your preferred contact details *(e.g. email/phone number).*

For peer review only
